# Mechanisms of Gills Response to Cadmium Exposure in Greenfin Horse-Faced Filefish (*Thamnaconus septentrionalis*): Oxidative Stress, Immune Response, and Energy Metabolism

**DOI:** 10.3390/ani14040561

**Published:** 2024-02-07

**Authors:** Xuanxuan Zhang, Wenquan Zhang, Linlin Zhao, Li Zheng, Bingshu Wang, Chengbing Song, Shenghao Liu

**Affiliations:** 1 School of Advanced Manufacturing, Fuzhou University, Jinjiang 362200, China; zhangxuanxuan930@163.com (X.Z.); zhengli@fio.org.cn (L.Z.); bswang@fzu.edu.cn (B.W.); 2 Key Laboratory of Marine Eco-Environmental Science and Technology, First Institute of Oceanography, Ministry of Natural Resources, Qingdao 266061, China; zhaolinlin@fio.org.cn; 3 National Deep Sea Center, Ministry of Natural Resources, Qingdao 266061, China; zhangwq@ndsc.org.cn

**Keywords:** cadmium stress, transcriptomics, metabolomics, oxidative stress, energy metabolism disorder, immune response

## Abstract

**Simple Summary:**

Cadmium (Cd) is one of the most prevalent and hazardous heavy metals. Marine Cd pollution has become a global issue, and the impact of Cd stress on marine organisms is of particular concern. Factors such as the duration and concentration of Cd exposure, as well as the species and life stage of fish, can all influence the severity of its harmful effects. To gain a comprehensive understanding of response mechanisms of commercial fishes to Cd stress, we chose greenfin horse-faced filefish (*Thamnaconus septentrionalis*) as study species. Our study provided new insights into the toxic effects and signal transduction/metabolic pathways of Cd exposure in marine fishes.

**Abstract:**

Cadmium (Cd) pollution has become a global issue due to industrial and agricultural developments. However, the molecular mechanism of Cd-induced detrimental effects and relevant signal transduction/metabolic networks are largely unknown in marine fishes. Here, greenfin horse-faced filefish (*Thamnaconus septentrionalis*) were exposed to 5.0 mg/L Cd up to 7 days. We applied both biochemical methods and multi-omics techniques to investigate how the gills respond to Cd exposure. Our findings revealed that Cd exposure caused the formation of reactive oxygen species (ROS), which in turn activated the MAPK and apoptotic pathways to alleviate oxidative stress and cell damage. Glycolysis, protein degradation, as well as fatty acid metabolism might assist to meet the requirements of nutrition and energy under Cd stress. We also found that long-term (7 days, “long-term” means compared to 12 and 48 h) Cd exposure caused the accumulation of succinate, which would in turn trigger an inflammatory response and start an immunological process. Moreover, ferroptosis might induce inflammation. Overall, Cd exposure caused oxidative stress, energy metabolism disturbance, and immune response in greenfin horse-faced filefish. Our conclusions can be used as references for safety risk assessment of Cd to marine economic fishes.

## 1. Introduction

Cadmium (Cd), one of the most prevalent and hazardous heavy metals, exhibits an uneven distribution in the ocean, with higher content typically found in coastal waters and sediment [1]. In recent years, due to the development of industries such as mining and electroplating, the discharge of Cd-containing wastewater has led to an accumulation of Cd in rivers and coastal waters. For example, a study based on the content and distribution characteristics of heavy metal elements in Bohai sediment found that Cd posed a significant ecological risk, with the highest enrichment level among heavy metals caused by human activities [2]. Additionally, deep-sea mining has become a future trend. However, mining operations inevitably lead to the release of heavy metals such as copper, Cd, zinc, lead, and iron, which can cause excess heavy metals in seawater [3]. Marine Cd pollution has become a global issue, and the impact of Cd stress on marine organisms is of particular concern [4].

Cd tends to accumulate significantly in the food chain, increasing the risk and toxic effects of Cd exposure in humans and other predators that consume fish containing Cd [5]. Once absorbed by an organism, Cd will reach various tissues and organs via the bloodstream and enter cells through calcium-ion channels, inhibiting normal metabolism and causing tissue cell or organ structure damage. For example, upon exposure to 5, 10, and 20 µg/L of Cd, zebrafish (*Danio rerio*) showed lipid peroxidation and apoptosis [6]. Cd exposure of 0.125 mg/L for 21 d impaired antioxidant defenses and intestinal function in mud crab (*Scylla paramamosain*) [5]. Moreover, Cd exposure of 0.26 mg/L for 30 d caused immunosuppression, oxidative stress, and carbohydrate metabolism disorder in common carp (*Cyprinus carpio*) [7]. It is worth noting that, in contrast to freshwater species, the mechanisms underlying Cd-induced detrimental effects on marine fishes, particularly Cd-induced signaling networks, are still poorly understood. Factors such as the duration and concentration of Cd exposure, as well as the species and life stage of the fish, can all influence the severity of its harmful effects [8].

Greenfin horse-faced filefish (*Thamnaconus septentrionalis*, hereafter “filefish”) is widely dispersed in the Indo-West Pacific Ocean. Its meat is high in protein content with a pleasant taste. The meat, liver, and skin have therapeutic properties. Thus, filefish has become a significant economic species in China, Japan, and the Korean Peninsula [9]. Due to marine environmental pollution and overfishing, the wild filefish population has declined. Recently, research on filefish mainly focused on fishery resource assessments, population structure and distribution, reproductive behavior, feeding habits, artificial breeding methods, and early embryonic development [10]. There have been limited studies on the filefish’s defenses against marine heavy metal pollution [11,12].

Marine fishes tend to prioritize ion regulation over metal detoxification when exposed to heavy metals. They employ strategies such as reducing metal absorption from water, minimizing metal accumulation in gills, and enhancing ion transporters to maintain osmotic balance [13]. In addition, gill tissues play crucial roles in their physiology and serve as a primary site for various physiological processes. Understanding the mechanisms of gills response to heavy metal toxicity is crucial for assessing the impact of pollution on marine ecosystems and developing strategies to mitigate its harmful effects. Therefore, this study was set out to uncover the Cd-induced detrimental effects on gill tissues and underlying mechanism of signal transduction/metabolic pathways through a multi-omics method.

## 2. Materials and Methods

### 2.1. The Cultivation of Filefish

A total of 110 adult filefish were donated by Qingdao Haichang Polar Ocean World (Qingdao, China). These filefish were six months old, with a body length of 15 ± 2 cm and a weight of 100 ± 5 g. They were raised in water tanks (72.2 cm × 52.5 cm × 43.3 cm, 110 L) that were equipped with inflatable filtration systems. They were acclimatized to laboratory conditions for two weeks with a temperature of 20 ± 1 °C, salinity 31 ± 1, pH 8 ± 0.1, and dissolved oxygen (DO) 7.5 ± 1.0 mg/L on a 12/12 light/dark cycle (light on at 8:00 a.m.). Filefish were fed with shrimp three times a day at about 3% of its body weight (i.e., 3 ± 0.15 g) to maintain normal vital activities. Additionally, the seawater was entirely replaced every day. This research was approved by the Committee on the Ethics of Animal Experiments of First Institute of Oceanography, MNR.

### 2.2. Adult Filefish Exposure Experiment

The concentration of Cd was determined based on previous studies and our preliminary experiment. The LC_50_ (half lethal concentration) of Cd for 96 h treatment was 9.19 mg/L in juvenile filefish [12]. In addition, treatment concentrations that exceeded environmental regulations were used to force organisms to produce strong stress responses and to ensure that the results were meaningful [1,14,15]. For example, in a study exploring effects of Cd on sea cucumber (*Apostichopus japonicus*), the maximum concentration in the Cd-treated group was set to 50 mg/L [14]. Therefore, to investigate the response of filefish to Cd stress, CdCl_2_·2.5H_2_O (purity: AR, Sinopharm Shanghai Co., Ltd., Shanghai, China) was added to seawater to reach a concentration of 5.0 mg/L as well.

**Figure 1:**
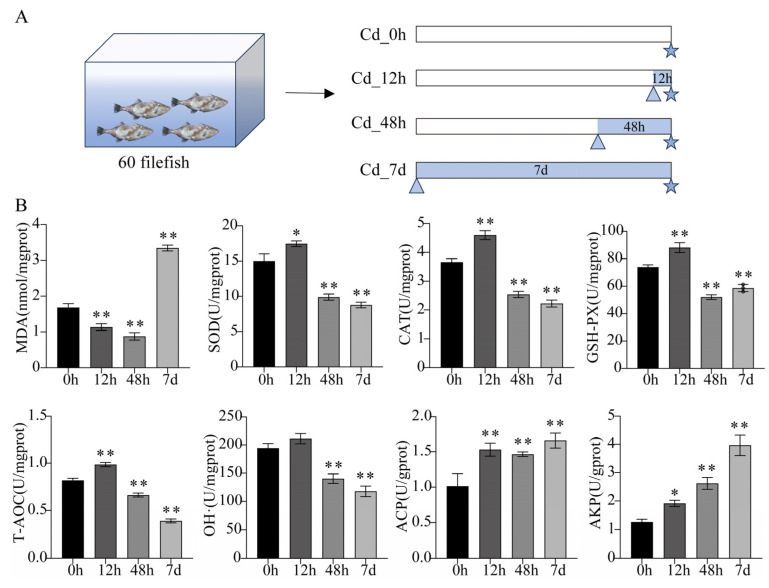
Experimental setting and physiological and biochemical measurements. (**A**) Diagrammatic sketch illustrating the experimental set-up and sampling strategy of the present study. Rectangles, triangles, and pentagrams indicate the experimental period after domestication, the start time of Cd stress, and the sampling time, respectively. (**B**) The biochemical features of filefish under Cd stress. U in enzyme indicators represents its activity, while in non-enzyme indicators, U represents the corresponding capacity. U/(m)gprot means enzyme activity or corresponding capacity in per (milli)gram of tissue protein. nmol/mgprot represents the concentration of MDA per milligram of tissue protein, with the unit in nanomoles. Values represent the mean ± standard deviation (n = 3). Significant difference was marked with asterisk (* *p* < 0.05, ** *p* < 0.01).

After two weeks of domestication, 60 filefish were randomly selected from 110 filefish for biochemical parameters assay, transcriptome sequencing, and quantitative real-time PCR (qPCR) analyses. These fish were randomly divided into 12 tanks, with 5 fish in each tank. ICP-MS (Inductively coupled plasma-Mass Spectrometry) was used to measure actual Cd concentration in tanks. McRae et al. (2018) described the specific process in detail [16]. Fish in the treatment groups were exposed to Cd-contaminated seawater at actual concentrations of 5.0 ± 0.1, 5.0 ± 0.2, and 5.0 ± 0.2 mg/L for 12 h (Cd_12h), 48 h (Cd_48h), and 7 d (Cd_7d), respectively, while the control group fish (Cd_0h) were kept in clean seawater. Three replicate tanks were set up for each group. Filefish were stopped feeding 24 h prior to sampling. There were 3 individuals (without gender ratio) randomly collected and dissected for gill tissues from each tank. Then, they were mixed into one sample. Such a sample served as a single biological replicate. To avoid the effect of circadian rhythm, each group was sampled at 9:00 a.m. (Figure 1A). Considering the welfare of animals, the fish were anesthetized with MS-222 (100 ppm, Sigma, St. Louis, MO, USA). In addition, another 50 filefish were chosen from 110 filefish for metabolomic analysis. These fish were randomly divided into two groups of 5 parallel tanks of 5 fish each. The actual Cd concentration in the treatment group was 5 ± 0.3 mg/L, and the treatment time was 7 d. Sampling methods were consistent with those described above. There were no fish deaths throughout the experiment, except for sampling.

### 2.3. Detection of Biochemical Parameters

To prepare the gill tissues of filefish under Cd stress (0 h, 12 h, 48 h, and 7 d) for biochemical parameter detection, the collected gill tissues were rinsed and homogenized with pre-cooled physiological saline (1:9 *w/v*). Then, the supernatant obtained by centrifugation (5000× *g*, 10 min, 4 °C) was used for biochemical assays according to the commercial kit instructions (Nanjing Jiancheng Bioengineering Institute, Nanjing, China), including malondialdehyde (MDA) content, superoxide dismutase (SOD) activity, catalase (CAT) activity, glutathione peroxidase (GSH-PX) activity, hydroxyl radical (OH·) scavenging ability, total antioxidant capacity (T-AOC) ability, acid phosphatase (ACP) activity, and alkaline phosphatase (AKP) activity. Each experiment was replicated as three biological replicates and three technical replicates.

### 2.4. RNA Extraction and Transcriptome Analysis

Total RNA was extracted from gill tissues of Cd-treated filefish (0 h, 12 h, 48 h, and 7 d) using TRIzol reagent (Invitrogen, Carlsbad, CA, USA). The transcriptome sequencing process was performed according to the steps we described previously [17]. Libraries were sequenced on Illumina novaseq 6000 Platform for 6G raw data and generated 150nt pair-end reads. We used HISAT2 v2.0.4 to align quality-controlled clean reads to reference genome [18]. HTSeq v0.6.1 was used to count the reads numbers mapped to each gene. Then, FPKM (expected number of Fragments Per Kilobase of transcript sequence per Millions base pairs sequenced) of each gene was calculated based on the length of the gene and reads count mapped to this gene [19]. Differential expression analysis of two groups was performed using the DESeq R package v1.10.1 [20]. Differential expression genes (DEGs) were screened with a threshold of adjusted *p*-value < 0.05. Gene Ontology (GO) enrichment analysis of DGEs was implemented by the GOseq R package, while KEGG (Kyoto Encyclopedia of Genes and Genomes) pathway enrichment analysis was conducted with KOBAS v3.0 software. A corrected *p*-value < 0.05 was considered significantly enriched in GO terms and KEGG pathways.

### 2.5. Metabolome Analysis

The gill tissues from the Cd-treated group (7 d) were collected for metabolomic analysis considering that the accumulation of metabolites requires some time. Metabolite extraction and detection were performed in accordance with previously described methods [21]. Briefly, samples were ground to powder with liquid nitrogen using a mixer mill (MM 400, Retsch, Laichi, Germany). Then, 20 mg of powder for each sample was extracted with 400 μL of aqueous methanol (Methanol/Water = 7:3, *v*/*v*) containing internal standard and shaken at 2500 rpm for 5 min. After placing on ice for 15 min, the extract was centrifuged at 12,000× *g* for 10 min (4 °C). Supernatant (300 μL) was collected and placed in −20 °C for 30 min. The supernatant was collected and transferred to vials for UPLC-QTOF-MS analysis (UPLC, ExionLC AD, https://sciex.com.cn/ (accessed on 21 February 2023); MS, QTRAP^®^ System, https://sciex.com/ (accessed on 21 February 2023)). Significantly changed metabolites (SCMs) were identified with a threshold of VIP values (variable importance in project, VIP > 1) and *p*-values (*p*-value < 0.05, Student’s *t* test). These metabolites were annotated using the KEGG Compound database and mapped to the KEGG Pathway database for pathway analysis.

### 2.6. Quantitative Real-Time RT-PCR Analysis

To verify expression levels of DEGs in the transcriptome, ten DEGs were randomly selected from the combined transcriptome and metabolome analyses for quantitative real-time RT-PCR (qPCR). Specific primers are shown in Table S1, with β-tublin serving as a reference gene. Xiang et al. (2023) reported the details of the qPCR protocol and calculation [22]. This experiment was carried out in three biological replicates and three technical replicates.

### 2.7. Statistical Analysis

The biochemical assay and qPCR data were analyzed with GraphPad Prism 9.0 software (GraphPad Software Inc., San Diego, CA, USA). One-way analysis of variance (ANOVA) was used to evaluate differences in the means of the data. Levene’s test and the Kolmogorov–Smirnov test were used to assess the data for homogeneity and normality before performing ANOVA. Results were expressed as mean ± standard deviation (S.D.). Statistical significance between the experimental and control groups was denoted by asterisks (*), where * *p* < 0.05 and ** *p* < 0.01. Mechanism maps were drawn by Adobe Illustrator 2021 software (Adobe Systems Incorporated, San Jose, CA, USA).

## 3. Results

### 3.1. Biochemical Parameters Analysis of Filefish Gill Tissues after Cd Exposure

MDA content was first significantly decreased and then significantly increased with long-term (7 d) exposure to Cd stress. Compared to the control, the activities of SOD, CAT, and GSH-PX were significantly reduced at 48 h and 7 d of Cd stress, while all were enhanced at 12 h. T-AOC capacity and the ability to inhibit OH· production showed a similar trend. Additionally, the activities of ACP and AKP were significantly increased in three Cd-treated groups (Cd_12h, Cd_48h, and Cd_7d), with AKP activity showing a time-dependent increase, reaching its maximum at 7 d (Figure 1B). Therefore, within a short period of time, filefish responded to Cd stress by enhancing antioxidant enzyme activities.

### 3.2. Transcriptome Analysis of Filefish Gill Tissues after Cd Exposure

We evaluated the data output quality of quantitative transcriptome sequencing (Table S2). After quality trimming, a total of 86.37 Gb clean reads were generated from 12 samples. UMI labeled reads constituted 92.22–95.65% of clean reads. The Q30 percentage exceeded 92.35%, and average GC content was 51.24%. Moreover, deduplicated UMI reads accounted for 61.80–68.23% of the total UMI reads aligned to the reference genome. The average mapping rate of UMI clean reads to filefish genome reached 85.57% (Table S3). These findings collectively suggested that the transcriptome data were of high quality.

**Figure 2:**
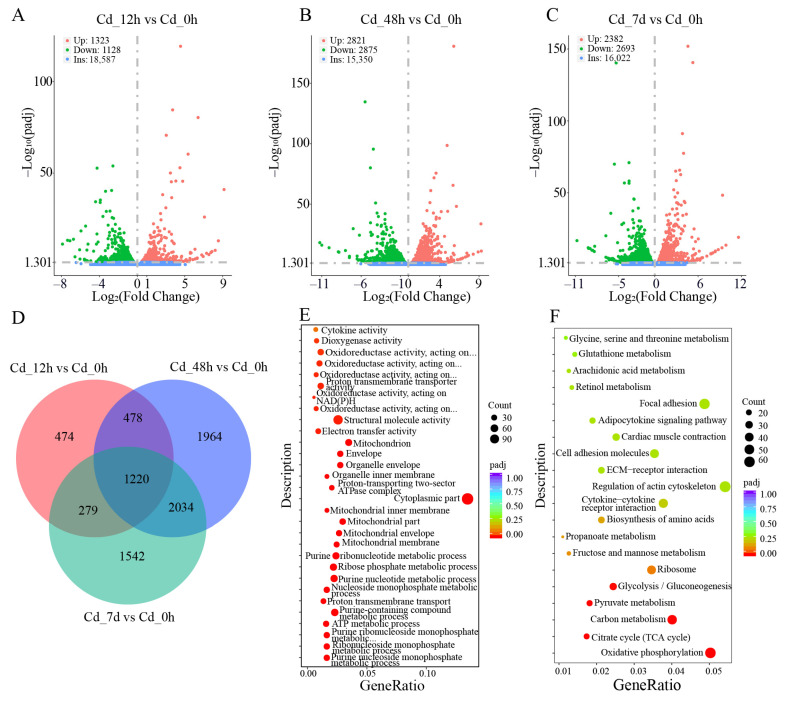
DEGs, GO term, and KEGG pathway analysis of filefish exposed to Cd. (**A**–**C**) DEGs volcano map of each comparison group. (**D**) The Venn diagram shows the number of shared and unique DEGs in the comparison groups. (**E**) The top 30 enriched GO terms based on DEGs of the Cd_7d group. (**F**) The top 20 enriched KEGG pathways based on DEGs of the Cd_7d group. Bubble size is proportional to the number of DEGs enriched in each of the pathways, and bubble color denotes the degree of significance, from the highest (red) to the lowest (purple). Gene Ratio represents the ratio of the number of DEGs to the total number of annotated genes in this pathway.

There were 2451 (1323 up- and 1128 down-regulated), 5696 (2821 up- and 2875 down-regulated), and 5075 (2382 up- and 2693 down-regulated) DEGs in Cd_12h vs. Cd_0h, Cd_48h vs. Cd_0h, and Cd_7d vs. Cd_0h, respectively (Figure 2A–C). A Venn diagram showed that 1220 DEGs were shared among three Cd-treated groups (Figure 2D). These results indicated that a large number of genes in filefish responded to Cd stress.

Subsequently, GO functional enrichment analysis was conducted on the DEGs obtained from different stress groups. Molecular function (MF), biological processes (BP), and cellular composition (CC) displayed the top 10 significantly enriched GO terms (Figure 2E and Figure S1A,B). For example, Rho GTPase binding, GTPase binding, and structural constituent of ribosome were enriched in MF, intracellular signal transduction, lipid catabolic process, and peptide metabolic process in BP, polymeric cytoskeletal fiber, supramolecular complex, ribosome, and cytoplasmic part in CC. Particularly, for the Cd_7d vs. Cd_0h group, electron transfer activity and oxidoreductase activity were significantly enriched in MF, purine nucleoside monophosphate metabolic process and ATP metabolic process in BP, mitochondrial membrane and mitochondrion in CC. GO functional enrichment analysis showed that Cd exposure can activate the activity of some catalytic enzymes in filefish, as well as redox processes in mitochondria, thereby improving the tolerance to Cd stress.

To further reveal the cellular metabolic pathways involved in DEGs, we compared all DEGs to KEGG metabolic pathways and mapped 20 significant specific pathways (Figure 2F and Figure S1C,D). Table S4 showed the representative KEGG pathways associated with Cd stress, such as ferroptosis, apoptosis, lysosome, MAPK signaling pathway, glycolysis/gluconeogenesis, fatty acid metabolism, TCA cycle, and adipocyte cytokine signaling pathway. DEGs were mainly clustered in metabolic pathways associated with “Oxidative stress, oxidative damage and apoptosis”, “Energy metabolism”, and “Immune response”.

### 3.3. Metabolomics Analysis of Filefish Gill Tissues after Cd Exposure

**Figure 3:**
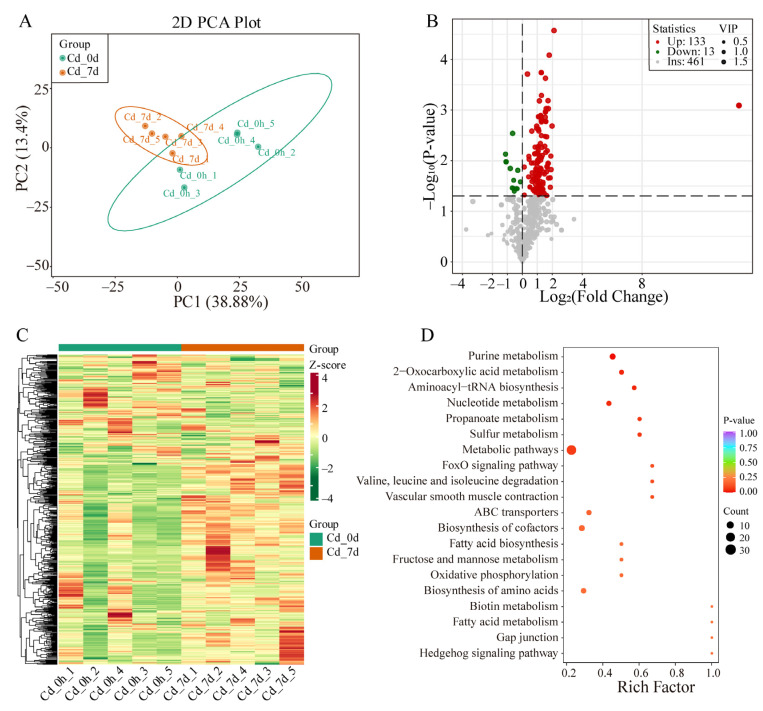
Multivariate statistical analysis of metabolites in filefish. (**A**) Principal clustering analysis (PCA). (**B**) SCMs volcano map. (**C**) Hierarchical clustering analysis (HCA). (**D**) The top 20 enriched KEGG pathways based on SCMs of Cd_7d group.

To investigate metabolic changes in the gill tissues of filefish following Cd exposure, UPLC-MS/MS was used for metabolite detection. Principal component analysis (PCA) was conducted to analyze the data, and the first two principal components (PC1 and PC2) accounted for 38.88% and 13.4% of variance, respectively. The PCA score plot revealed clear separation between the Cd_0h and Cd_7d, with each biological replicate clustering together, indicating high reproducibility and reliability of the experiment (Figure 3A). Additionally, an OPLS-DA model was applied to compare metabolite contents and identify variables responsible for group differences. The R^2^X, R^2^Y, and Q^2^ parameters were calculated to assess model stability and reliability, with higher values indicating better performance. A Q^2^ of >0.5 was considered effective. The OPLS-DA model revealed significant metabolic phenotype differences between the Cd_7h group and Cd_0h group with R^2^X, R^2^Y, and Q^2^ values of 0.632, 0.999, and 0.665, respectively (Figure S2A,B). These results indicated that Cd exposure significantly altered metabolite profiles in the gill tissues of filefish.

A total of 146 SCMs (133 up- and 13 down-regulated) were detected (Figure 3B). A heat map showed the overall change in SCMs (Figure 3C). Particularly, most SCMs were secondary metabolites, including oxidized lipids, organic acid and its derivatives, small peptide, acylcarnitine, nucleotide and its metabolites, and amino acid and its metabolites (Table S5). KEGG metabolic pathway analysis showed that SCMs were enriched in the pathways of fatty acid metabolism, fructose and mannose metabolism, propanoate metabolism, ABC transporters, Valine, leucine and isoleucine degradation, biosynthesis of amino acids, and others (Figure 3D).

### 3.4. Response Mechanism Analysis of Filefish Gill Tissues after Cd Exposure

To further understand response mechanisms to Cd stress in filefish, we conducted a comprehensive analysis combining transcriptomics and metabolomics. As mentioned above, there was a significant enrichment of DEGs and SCMs for JNK and p38 MAPK, glycolysis, fatty acid degradation, and the TCA cycle pathway in KEGG analysis. This gave us clues that they were the main response pathways.

**Figure 4:**
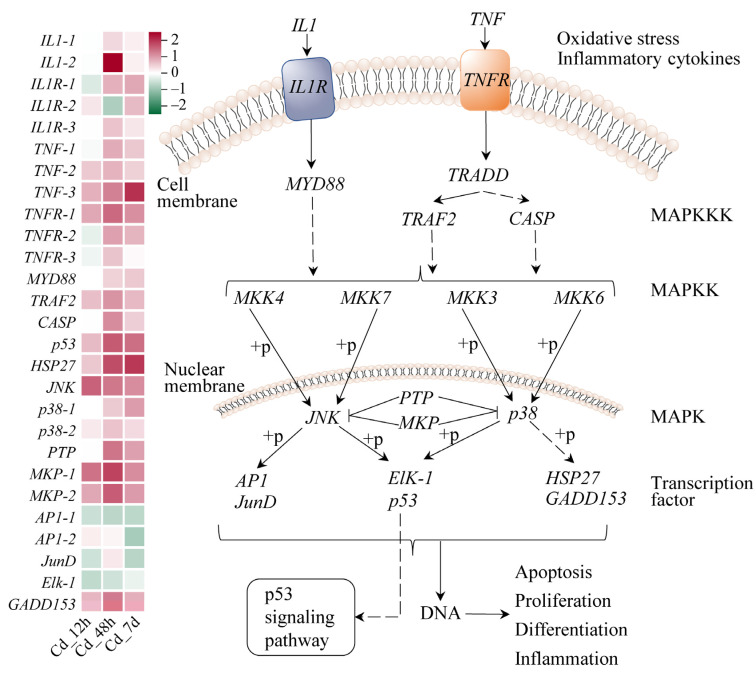
JNK and p38 MAPK signaling pathway of filefish upon Cd exposure. Genes are shown in italic to distinguish with metabolites. The heat map indicates relative gene expression in the Cd-stressed group compared to Cd_0h, which was set to 0. SCMs expressions are shown as red (upregulation) or green (downregulation). The following mechanism diagrams are similar. Note: *IL1*: interleukin 1, *IL1R*: interleukin 1 receptor, *TNF*: tumor necrosis factor, *TNFR*: tumor necrosis factor receptor, *MYD88*: myeloid differentiation factor 88, *TRAF2*: TNF receptor-associated factor 2, *CASP*: Caspase, *HSP27*: heat shock protein 27, *JNK*: Jun N-terminal protein kinase, *MKP*: mitogen-activated protein kinase phosphatase, *GADD153*: growth arrest- and DNA damage-inducible gene 153.

In the JNK and p38 MAPK signaling pathway, we discovered 27 DEGs (Figure 4). Factors associated with immunity and inflammation interleukin 1 (*IL1*), interleukin 1 receptor (*IL1R*), tumor necrosis factor (*TNF*), and tumor necrosis factor receptor (*TNFR*) showed increased levels of gene expression. Notably, expression levels of genes encoding two key protein kinases, Jun N-terminal kinase (*JNK*) and *p38*, were both upregulated in this pathway. The JNK transcript level reached its peak at 12 h, exhibiting a Log_2_(Fold Change) of approximately 1.34. On the other hand, the gene expression levels of *p38-1* increased over time, reaching its maximum at 7 d, while *p38-2* showed the highest abundant at 48 h, with a Log_2_(Fold Change) of 0.52. Expression levels of *AP1*, *JunD*, and *Elk-1*, which are associated with cell proliferation and differentiation, were decreased at 7d. In contrast, transcription factor growth arrest- and DNA damage-inducible gene 153 (*GADD153*), responsible for regulating genes related to the apoptotic pathway, exhibited a significant upregulation at different time points and peaked at 48 h. The expression level of heat shock protein 27 (*HSP27*) increased in a time-dependent manner, reaching a maximum at 7 d.

**Figure 5:**
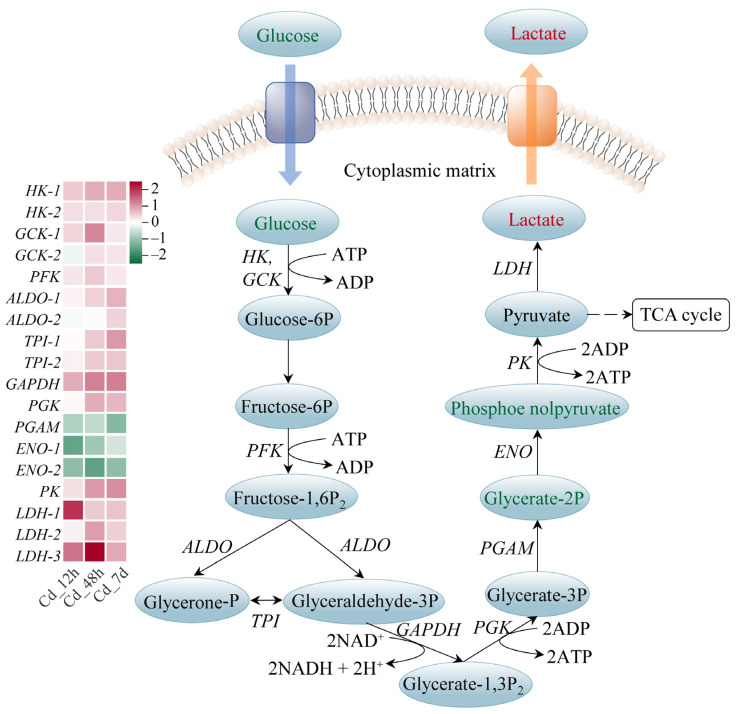
Glycolysis pathway of filefish upon Cd exposure. Note: *HK*: hexokinase, *GCK*: glucokinase, *PFK*: phosphofructokinase, *ALDO*: aldolase, *TPI*: triose-phosphate isomerase, *GAPDH*: glyceraldehyde-3-phosphate dehydrogenase, *PGK*: phosphoglycerate kinase, *ENO*: enolase, *PGAM*: phosphoglycerate mutase, *PK*: pyruvate kinase, *LDH*: lactate dehydrogenase.

**Figure 6:**
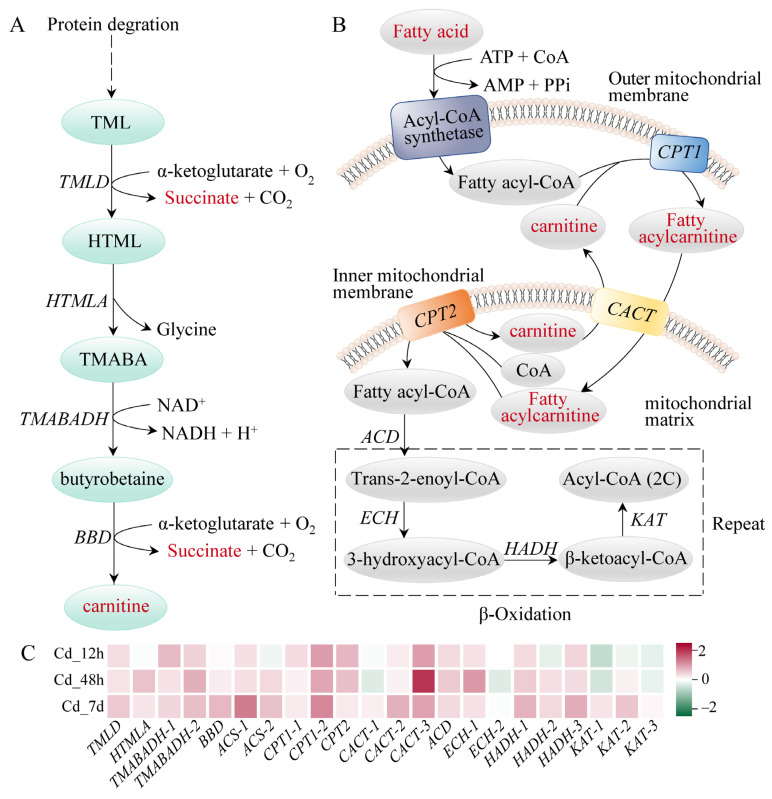
Fatty acid degradation-related pathways of filefish upon Cd exposure. (**A**) Carnitine biosynthetic pathway, (**B**) Fatty acid β-oxidation process, (**C**) The expression of relevant genes after Cd exposure. Note: *TMLD*: trimethyllysine dioxygenase, *HTMLA*: 3-hydroxy-6-N-trimethyllysine aldolase, *BBD*: γ-butyrobetaine dioxygenase, *TMABADH*: 4-N-trimethylaminobutyraldehy dehydrogenase, *ACS*: Acyl-CoA synthetase, *CPT1*: carnitine palmitoyltransferase1, *CPT2*: carnitine palmitoyltransferase2, *CACT*: carnitine acylcarnitine translocase, *ACD*: Acyl-CoA dehydrogenase, *ECH*: Enoyl-CoA hydratase, *HADH*: 3-hydroxyacyl-CoA dehydrogenase, *KAT*: β-ketoacyl-CoA thiolase.

The majority of enzymes involved in the glycolysis pathway had higher gene expression levels; examples include hexokinase (*HK*), aldolase (*ALDO*), triose-phosphate isomerase (*TPI*), and pyruvate kinase (*PK*), which showed a time-dependent rise. Notably, lactate, the final product of this pathway, showed significant accumulation with a Log_2_(Fold Change) of 0.99 (Figure 5). Carnitine can be biosynthesized from methionine and lysine during the degradation of proteins. We found that the expressions of the enzymes related to carnitine biosynthesis were markedly increased upon Cd exposure. Meanwhile, 14 carnitine analogues exhibited a significant accumulation (Figure 6A). Furthermore, expression levels of carnitine palmityl transferase 1 (*CPT1*), carnitine palmityl transferase 2 (*CPT2*), Acyl-CoA dehydrogenase (*ACD*), and 3-hydroxyacyl-CoA dehydrogenase (*HADH*) were all increased upon Cd exposure (Figure 6B,C).

**Figure 7:**
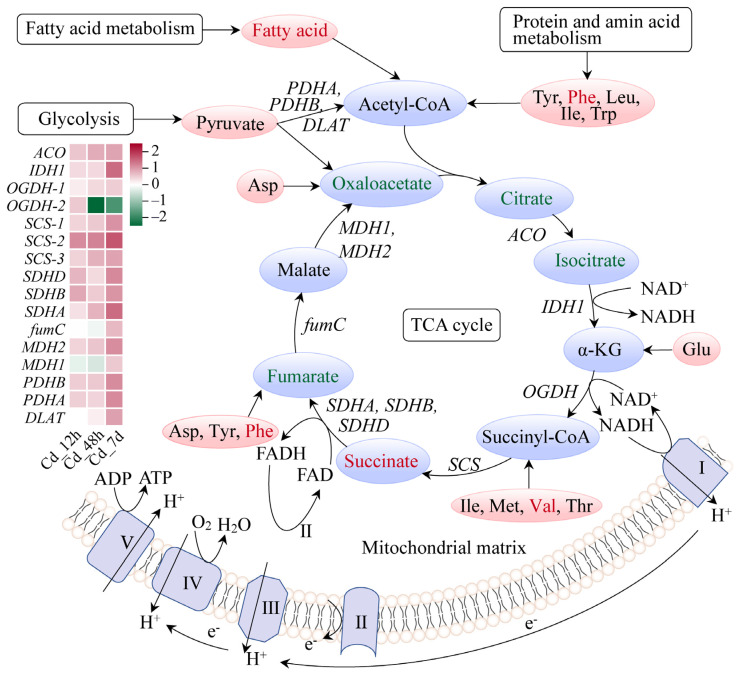
Citrate cycle (TCA cycle) pathway of filefish upon Cd exposure. Note: *ACO*: aconitase, *IDH1*: isocitrate dehydrogenase 1, *OGDH*: oxidative glutarate dehydrogenase, *SCS*: succinyl-CoA synthetase, *SDH(A/B/C/D)*: succinate dehydrogenase, *fumC*: fumarateC, *MDH*: malate dehydrogenase, *PDH(A/B)*: pyruvate dehydrogenase, *DLAT*: dihydrolipoamide S-acetyltransferase.

Most TCA cycle pathway metabolites, including oxaloacetate, citrate, and isocitrate, were downregulated under long-term Cd stress conditions. Interestingly, succinate, acting as an immune metabolite, was significantly accumulated, with a Log_2_(Fold Change) of 1.31. Additionally, tyr, vale, and phenylalanine significantly accumulated across the TCA pathway. Gene expression levels of enzymes involved in the TCA cycle pathway were largely enhanced (Figure 7). These results further indicated that Cd has exerted extensive effects on gene–metabolite networks in the gills of filefish.

Gene expression levels of ten DEGs in transcriptome sequencing were verified and are shown in Figure S3. qPCR analysis showed that Cd stress significantly increased gene expression levels of *TNF-3*, *JNK*, *GADD153*, *LDH-3*, *PK*, *TMLD*, *CACT-3*, *SDHA*, and *SCS-2*, while it significantly decreased gene expression level of *ENO-2*. There were consistent trends in gene expression levels from transcriptome and qPCR analyses. For example, qPCR analysis showed that *TNF-3* and *JNK* gene expression levels in the JNK/p38 MAPK signaling pathway in the Cd_7d group were both significantly upregulated and were 10.19-fold and 6.19-fold higher than those in the Cd_0h group, respectively. Correspondingly, gene expression levels of these two genes were also significantly increased with 4.83-fold and 1.96-fold in the Cd_0h group in transcriptome analysis. These findings indicated that sequencing results were reliable and reproducible.

## 4. Discussion

To gain a comprehensive understanding of response mechanisms of commercial fishes to Cd stress, filefish were exposed to 5.0 mg/L of Cd up to 7 d, and the responses of gill tissues were then evaluated using both biochemical methods and multi-omics techniques. Our findings revealed that (1) Cd exposure activated the JNK and p38 MAPK signaling pathway, energy metabolism disorder, and immune response in marine economic fishes based on transcriptomic and metabolomic analyses; (2) Long-term Cd exposure caused the accumulation of metabolites like lactate and succinate, indicating that filefish was experiencing anaerobic energy metabolism; (3) We also suggested that succinate, a tricarboxylic acid (TCA) cycle intermediate, would drive the inflammatory responses upon Cd exposure and promote innate immunological process. Collectively, our study revealed Cd-induced detrimental effects on gill tissues and the underlying mechanism of signal transduction/metabolic pathways. Furthermore, the amount of lactate, carnitine, and succinate reflected the degrees of damage to adult filefish, which can be used as references for safety risk assessment.

### 4.1. Cd Exposure Induced Oxidative Stress and Apoptosis in Gills

The MAPK signaling pathway regulates various biological processes, such as cell proliferation, apoptosis, and stress response. Within this pathway, JNK and p38 branches are important for stress signal transduction, helping cells cope with oxidative pressure and protecting their functionality and integrity by regulating cell survival and apoptosis, antioxidant response, and generation of inflammatory factors [23]. As mentioned above, the JNK and p38 MAPK signaling pathway was enhanced upon Cd exposure, as were expression levels of IL1 and TNF-α (Figure 4). Previous studies have shown that exposure to Cd activated the *JNK*, *Erk1/2*, and *p38* pathways in rat and human cells [24]. Similarly, Cd and arsenite activated the JNK/p38 MAPK and ERK signaling pathway in drosophila through oxidative stress [25]. In marine metazoans, Cd exposure increased the expressions of genes related to immune factors including TNF and Toll-like receptor signaling pathways [26]. When cells encounter oxidative stress, JNK and p38 are activated and mediate cellular stress response by phosphorylating a series of downstream target proteins. For example, JNK can regulate the expression of antioxidant response genes, enhancing cells’ ability to adapt to oxidative stress [27], while p38 can phosphorylate various transcription factors and protein kinases, promoting the production of inflammatory factors and expression of genes related to cell stress response [28]. This study found that under various conditions, apoptosis-promoting transcription factors *p53* and *GADD153*, along with *HSP27* which has immunomodulatory effects and increased antioxidant capacity, were all enhanced. However, *AP1*, *JunD*, and *Elk-1*, which are associated with cell proliferation and differentiation, were reduced (Figure 4). Therefore, we speculated that Cd exposure activated the JNK and p38 signaling pathway and apoptosis pathway. The upregulation of genes on these pathways may represent an adaptive response of filefish to Cd exposure.

Oxidative stress is a critical biological process observed in aquatic organisms when they encounter various environmental challenges, such as exposure to heavy metals, toxins, and radiation [29]. It arises from an imbalance between the generation and elimination of ROS. Overabundance of ROS can harm DNA, lipids, proteins, and other components of cells, resulting in oxidative damage and malfunction. Enzymatic antioxidants play a vital role in converting ROS into less harmful substances, including SOD, CAT, and GSH-PX. Exposure to Cd has been found to induce the elevation of ROS levels in aquatic organisms, leading to oxidative stress [30]. In this study, the activities of antioxidant enzymes, T-AOC, and OH· scavenging ability were increased in the early stages of Cd stress (Figure 1B). Consistent with our results, SOD and CAT activities were significantly enhanced when mud crabs were exposed to 0.01 mg/L of Cd [5]. Besides, carnitine was accumulated excessively in the gills of filefish upon Cd exposure (Figure 6). It has been demonstrated that carnitine possesses antioxidant and anti-inflammatory properties [31]. In mouse brain, Cd-induced mitochondrial autophagy reduced ROS through the accumulation of carnitine [32]. Carnitine treatment could lessen DNA damage, improve histopathological abnormalities, and increase the activities of antioxidant enzymes in male mice exposed to Cd-induced damage [33]. These results suggested that enhanced antioxidant enzymes activities and carnitine accumulation were effective in scavenging ROS induced by short-term (12 h) Cd stress.

In contrast, long-term Cd stress weakened the antioxidant defense of filefish, which in turn was unable to resist oxidative damage to nucleic acids, lipids, and proteins caused by excessive ROS. Under severe conditions, oxidative stress can cause oxidative damage to cells, ultimately leading to apoptosis [34]. The accumulation of ROS in cells triggers lipid peroxidation, which results in the formation of lipid peroxidation products like MDA. Therefore, MDA content can serve as an indicator of the level of oxidative damage in an organism [35]. Our study showed that after 7 d of Cd stress, the MDA content was significantly increased, indicating that long-term Cd stress induced lipid peroxidation (Figure 1B). Similar findings were found in crucian carp with 5 mg/L of Cd after 21 d and in zebrafish with 10 μg/L of Cd after 48 d [1,6]. Notably, lysosome and apoptosis pathways were enriched in KEGG enrichment analysis of transcriptome sequencing (Table S4). Lysosomes are organelles containing a variety of enzymes that play a critical role as “detergents” in cellular metabolism, which is essential for maintaining cellular homeostasis. In addition, lysosomes are important regulators of apoptosis and autophagy [36]. Taken together, we speculated that Cd exposure induced the formation of ROS, which can stimulate the JNK and p38 signaling pathway and apoptotic pathways. This could alleviate Cd-induced oxidative stress in a short period of time. However, long-term Cd stress on filefish caused lipid peroxidation and, ultimately, cellular damage and apoptosis.

### 4.2. Cd Exposure Caused Energy Metabolism Disorder in Gills

Energy metabolism is crucial for maintaining physiological functions and responding to stress in organisms. Lipids, sugars, and proteins, the main sources of energy, are metabolized mainly in the mitochondria. In general, the energy supply is sufficient for the organism’s needs, with excess energy being stored. Nevertheless, heavy metal stress can disrupt the energy balance of marine creatures, as the organism requires more energy to initiate stress responses, detoxification, and damage repair mechanisms [37].

Glycolysis is an important pathway of glucose metabolism. Glycolysis breaks down one molecule of glucose into two molecules of pyruvate, producing a net of two molecules of ATP and two molecules of NADH. Under anaerobic conditions, pyruvate is reduced to lactate, which contains most of the energy. In contrast, under aerobic conditions, pyruvate enters the mitochondria, where it is further metabolized through the TCA cycle and oxidative phosphorylation, producing large amounts of ATP. We found that gene expression levels of enzymes involved in glycolysis were significantly increased in the Cd-treated group, such as *HK*, *PK*, and *LDH* (Figure 5). This increase implied that glycolysis was promoted under Cd stress. Previous studies have found that the glycolysis pathway was significantly enhanced with increasing Cd concentration in mice [38]. In addition, we noted that *LDH* expression and lactate were upregulated. This was consistent with previous studies showing elevated plasma lactate in juvenile silver carp (*Hypophthalmichthys molitrix*) exposed to sublethal concentrations of Cd [39]. Lactate can be used as an indirect indicator of the fish’s response to stress, reflecting the fact that tissue demand for oxygen exceeded the supply [40]. We hypothesized that Cd stress might cause disturbances in the dynamic balance of body energy through anaerobic metabolic pathways.

Fatty acids exhibit higher energy storage efficiency compared to carbohydrates like glucose [37]. Carnitine plays a critical role in energy metabolism by facilitating the transport of long-chain fatty acids from cytoplasm to mitochondrial matrix and promoting β-oxidation of fatty acids [41]. In the present study, all carnitine compounds were accumulated in filefish gills. We also noted that the expressions of enzymes related to β-oxidation were elevated, such as *CPT1*, *HADH*, and *KAT* (Figure 6). These findings indicated that the oxidation process of fatty acids in gills was enhanced to meet energy demand under Cd stress. This phenomenon has also been demonstrated in hypo-salinity stressed hard clams (*Mercenaria mercenaria*) [37].

Proteins can be turned into energy through gluconeogenesis when the energy provided by fats and sugars cannot meet physiological needs. Based on transcriptomic analysis, the adipocyte cytokine signaling pathway was significantly enriched in all three experimental groups (Table S4). Leptin acts as a crucial regulator of energy intake and metabolic rate in this pathway. It exerts its anorexic effects by modulating neuropeptide levels through janus kinase (*JAK*), signal transducer and activator of transcription 3 (*STAT3*) phosphorylation, and nuclear transcription [42]. In our study, *STAT3* expression was upregulated, which may contribute to an anorexic situation in the organism, lacking exogenous substances for energy supply. Interestingly, some metabolites were significantly accumulated in the gills, such as most small peptides and lysine, norleucine, phenylalanine, and tryptophan (Table S5). We speculated that long-term Cd stress may stimulate protein and amino acid metabolism in filefish, producing more nutrients to meet energy requirements. This hypothesis aligned with previous findings where underwater noise stimulated carbohydrate and amino acid metabolism in sturgeon [43].

The TCA cycle is the final metabolic pathway for the three main nutrients (sugars, lipids, and proteins), yielding substantial energy production. We noted that gene expressions of almost all enzymes were upregulated in the TCA cycle pathway. Meanwhile, metabolomic analysis showed an excessive accumulation of free fatty acids and some small peptide molecules (Figure 7). This indicated that the TCA cycle pathway was activated and strengthened. Notably, succinate, an intermediate metabolite of the TCA cycle, was accumulated significantly in filefish. Excess succinate in the cytoplasm is a characteristic feature of anaerobic metabolism [44]. Cd exposure was reported to alter the abundance of metabolites associated with energy metabolism (ATP, AMP, phosphorylcholine, lactate, and succinate) mainly in the livers of halibut (*Paralichthys olivaceus*) [45]. Additionally, Cd exposure may cause a shift from aerobic to anaerobic energy metabolism [46]. These findings further confirmed our speculation that filefish predominantly relied on anaerobic metabolism after 7 d of Cd exposure.

### 4.3. Cd Exposure Triggered Cellular Inflammation and Activated Immune Response in Gills

Fishes belong to primitive jawed vertebrates, with relatively underdeveloped adaptive immunity and relatively weak immunological memory. Thus, innate immunity is particularly important for fishes to fight against pathogen infection. Inflammation is a physiological process that is vital to the host immune system’s defenses against infection or injury. Inflammation is triggered by pathogens, foreign objects, harmful chemicals, and the removal of necrotic cells [7]. Cd is a highly toxic heavy metal and an effective inflammatory stimulator. These toxic effects are accompanied by the activations of ferroptosis and other signal transduction/metabolic pathways [43]. Ferroptosis is a non-apoptotic cell death form characterized by excess iron aggregation and lipid peroxidation [47]. Cd exposure caused iron accumulation and activated ferroptosis-mediated inflammation. Meanwhile, Cd-induced liver toxicity was associated with ferroptosis, contributing to oxidative stress and the initiation of autophagy [43]. Our results revealed an enhanced ferroptosis-related metabolic pathway in fish gills upon Cd exposure (Table S4), highlighting the significance of ferroptosis in Cd-induced inflammation.

In addition, succinate has emerged as a critical regulator of innate immune responses in mammals, guiding macrophage effector functions by influencing the transcription of proinflammatory cytokines and the production of ROS [48]. Excessive succinate in mouse liver had been reported to induce inflammation by binding to succinate receptor 1 (*SUCNR1*) in stellate cell and macrophage populations, causing local activation of sucnr1-expressing macrophages and thereby initiating or intensifying the immune response [43]. We hypothesized that Cd stress caused succinate accumulation, driving an inflammatory response in gill tissues, which in turn activated immune responses in gill tissues of filefish.

ACP and AKP are important components of the innate immune system (i.e., non-specific immunity) and are widely present in lysosomes. It has been determined that the activities of ACP and AKP serve as appropriate indicators for assessing the immunity in polluted environments [49]. In this study, ACP and AKP activities were significantly raised in all treatment groups, consistent with Cd-treated crabs and wheat seedlings [50,51]. Our results indicated that filefish enhanced the immune process. A reduction in Cd-induced cellular damage was achieved by increasing the activities of ACP and AKP, as well as promoting the metabolism of phosphate in the organism.

## 5. Conclusions

Marine Cd pollution has become a global issue. However, the molecular mechanism of Cd-induced detrimental effects and relevant signal transduction/metabolic networks are largely unknown in marine fishes. The present study employed biochemical analysis, transcriptomics, and metabolomics to reveal the response of filefish gills to Cd exposure. In turn, this will lay the foundation for understanding the response mechanism of marine economic fishes to Cd stress. We found that MAPK signaling and ferroptosis pathways, as well as immune metabolites (particularly carnitine and succinate), might play critical roles in mediating the responses of filefish to Cd exposure. Furthermore, glycolysis, fatty acid degradation, TCA cycle, and protein and amino acid metabolic pathways were enhanced to ensure filefish’s nutritional and energy requirements under this adverse condition. Additionally, long-term Cd exposure increased the amounts of lactate and succinate, indicating that filefish underwent considerable anaerobic energy metabolism. Moreover, we also suggested that the accumulation of succinate would drive inflammatory response upon Cd exposure, then trigger innate immunological process. In conclusion, excessive Cd exposure may adversely affect filefish health by inducing oxidative damage, enhancing inflammatory response, and disrupting metabolic function. Our study offered new insights into detrimental effects and signal transduction/metabolic pathways upon Cd exposure in the gills of marine economic fishes.

## Data Availability

The raw sequence data reported in this paper have been deposited in the Genome Sequence Archive in the National Genomics Data Center, China National Center for Bioinformation/Beijing Institute of Genomics, Chinese Academy of Sciences (GSA: CRA011671) that are publicly accessible at https://ngdc.cncb.ac.cn/gsa (accessed on 4 July 2023).

## Supplementary Materials

The following supporting information can be downloaded at: https://www.mdpi.com/article/10.3390/ani14040561/s1, Figure S1: GO term and KEGG pathway analysis of filefish exposed to Cd; Figure S2: Multivariate statistical analysis of metabolites in filefish; Figure S3: Comparison of DEGs expression levels between qPCR and RNA-Seq; Table S1: Primer sequences for qPCR; Table S2: Summary of data quality in transcriptome sequencing; Table S3: The ratio of sequencing reads mapping to genome in different samples; Table S4: Representative pathways related to Cd stress of filefish; Table S5: Significantly changed metabolites in filefish after 7 d of Cd stress.

## References

[1] Wei W., Yang Q., Xiang D., Chen X., Wen Z., Wang X., Xu X., Peng C., Yang L., Luo M. (2023). Combined impacts of microplastics and cadmium on the liver function, immune response, and intestinal microbiota of crucian carp (*Carassius carassius*). Ecotoxicol. Environ. Saf..

[2] Wei Y., Ding D., Qu K., Sun J., Cui Z. (2022). Ecological risk assessment of heavy metal pollutants and total petroleum hydrocarbons in sediments of the Bohai Sea, China. Mar. Pollut. Bull..

[3] Zhou L., Li M., Zhong Z., Chen H., Wang M., Lian C., Wang H., Zhang H., Cao L., Li C. (2023). Toxicological effects of cadmium on deep-sea mussel Gigantidas platifrons revealed by a combined proteomic and metabolomic approach. Front. Mar. Sci..

[4] Liu W., Qiu H., Yan Y., Xie X. (2021). Acute Cd toxicity, metal accumulation, and ion loss in southern catfish (*Silurus meridionalis* Chen). Toxics.

[5] Cheng C.H., Ma H.L., Liu G.X., Fan S.G., Deng Y.Q., Jiang J.J., Feng J., Guo Z.X. (2023). Toxic effects of cadmium exposure on intestinal histology, oxidative stress, microbial community, and transcriptome change in the mud crab (*Scylla paramamosain*). Chemosphere.

[6] Hu W., Zhu Q.L., Zheng J.L., Wen Z.Y. (2022). Cadmium induced oxidative stress, endoplasmic reticulum (ER) stress and apoptosis with compensative responses towards the up-regulation of ribosome, protein processing in the ER, and protein export pathways in the liver of zebrafish. Aquat. Toxicol..

[7] Chen J., Xu Y., Han Q., Yao Y., Xing H., Teng X. (2019). Immunosuppression, oxidative stress, and glycometabolism disorder caused by cadmium in common carp (*Cyprinus carpio* L.): Application of transcriptome analysis in risk assessment of environmental contaminant cadmium. J. Hazard. Mater..

[8] Iftikhar N., Konig I., English C., Ivantsova E., Souders C.L., Hashmi I., Martyniuk C.J. (2023). Sulfamethoxazole (SMX) alters immune and apoptotic endpoints in developing zebrafish (*Danio rerio*). Toxics.

[9] Xu D., Liu K., Wang P., Chang Q., Chen S., Bian L., Liu C., Ge J. (2018). Analysis of nutritional composition in the muscle of *Thamnaconus septentrionalis*. Mar. Sci..

[10] Chirinos-Peinado D., Castro-Bedriñana J., Ríos-Ríos E., Mamani-Gamarra G., Quijada-Caro E., Huacho-Jurado A., Nuñez-Rojas W. (2022). Lead and cadmium bioaccumulation in fresh cow’s milk in an intermediate area of the Central Andes of Peru and risk to human health. Toxics.

[11] Wang B., Wang L., Wang A., Miao Y. (2021). Next-generation sequencing of the mitochondrial genome of *Thamnaconus septentrionalis* Gunther, 1877 (Aluteridae: Thamnaconus) specimen collected in China. Mitochondrial DNA Part B.

[12] Wang X., Bian L., Hu Q., Qin B., Chang Q., Ying N., Wu Y., Yang L., Chen S. (2023). Acute effects of cadmium on the antioxidant enzyme activities and histological structure of the gills and liver of juvenile *Thamnaconus septentrionalis*. Prog. Fish. Sci..

[13] Figueroa J.A., Stiner C.A., Radzyukevich T.L., Heiny J.A. (2016). Metal ion transport quantified by ICP-MS in intact cells. Sci. Rep..

[14] Zhang C., Lin C., Li L., Mohsen M., Wang T., Wang X., Zhang L., Huang W. (2023). Single and combined effects of microplastics and cadmium on the sea cucumber *Apostichopus japonicus*. Mar. Environ. Res..

[15] Nigro M., Bernardeschi M., Costagliola D., Della Torre C., Frenzilli G., Guidi P., Lucchesi P., Mottola F., Santonastaso M., Scarcelli V. (2015). n-TiO_2_ and CdCl_2_ co-exposure to titanium dioxide nanoparticles and cadmium: Genomic, DNA and chromosomal damage evaluation in the marine fish European sea bass (*Dicentrarchus labrax*). Aquat. Toxicol..

[16] McRae N.K., Gaw S., Glover C.N. (2018). Effects of waterborne cadmium on metabolic rate, oxidative stress, and ion regulation in the freshwater fish, inanga (*Galaxias maculatus*). Aquat. Toxicol..

[17] Liu S., Li T., Fang S., Zhang P., Yi D., Cong B., Zhang Z., Zhao L. (2022). Metabolic profiling and gene expression analyses provide insights into cold adaptation of an Antarctic moss *Pohlia nutans*. Front. Plant Sci..

[18] Bian L., Li F., Ge J., Wang P., Chang Q., Zhang S., Li J., Liu C., Liu K., Liu X. (2020). Chromosome-level genome assembly of the greenfin horse-faced filefish (*Thamnaconus septentrionalis*) using Oxford Nanopore PromethION sequencing and Hi-C technology. Mol. Ecol. Resour..

[19] Trapnell C., Williams B.A., Pertea G., Mortazavi A., Kwan G., van Baren M.J., Salzberg S.L., Wold B.J., Pachter L. (2010). Transcript assembly and quantification by RNA-Seq reveals unannotated transcripts and isoform switching during cell differentiation. Nat. Biotechnol..

[20] Wang L., Feng Z., Wang X., Wang X., Zhang X. (2010). DEGseq: An R package for identifying differentially expressed genes from RNA-seq data. Bioinformatics.

[21] Jiang W., Fang J., Du M., Gao Y., Fang J., Jiang Z. (2021). Integrated transcriptomics and metabolomics analyses reveal benzo[a]pyrene enhances the toxicity of mercury to the Manila clam, *Ruditapes philippinarum*. Ecotoxicol. Environ. Saf..

[22] Xiang J., Wu H., Gao J., Jiang W., Tian X., Xie Z., Zhang T., Feng J., Song R. (2023). Niclosamide exposure disrupts antioxidant defense, histology, and the liver and gut transcriptome of Chinese soft-shelled turtle (*Pelodiscus sinensis*). Ecotoxicol. Environ. Saf..

[23] Cho J.H., Kim D.H., Lee J.S., Seo M.-S., Kim M.E., Lee J.S. (2022). *Sargassum horneri (Turner) C. Agardh* extract regulates neuroinflammation in vitro and in vivo. Curr. Issues Mol. Biol..

[24] Chen L., Liu L., Luo Y., Huang S. (2007). MAPK and mTOR pathways are involved in cadmium-induced neuronal apoptosis. J. Neurochem..

[25] Ryabinina O.P., Subbian E., Iordanov M.S. (2006). D-MEKK1, the Drosophila orthologue of mammalian MEKK4/MTK1, and Hemipterous/D-MKK7 mediate the activation of D-JNK by cadmium and arsenite in Schneider cells. BMC Cell Biol..

[26] Strader M.E., Wong J.M., Hofmann G.E. (2020). Ocean acidification promotes broad transcriptomic responses in marine metazoans: A literature survey. Front. Zool..

[27] Shen H.-M., Liu Z.-g. (2006). JNK signaling pathway is a key modulator in cell death mediated by reactive oxygen and nitrogen species. Free Radic. Biol. Med..

[28] Dong X., Tang Y. (2022). Ntrk1 promotes mesangial cell proliferation and inflammation in rat glomerulonephritis model by activating the STAT3 and p38/ERK MAPK signaling pathways. BMC Nephrol..

[29] Kryston T.B., Georgiev A.B., Pissis P., Georgakilas A.G. (2011). Role of oxidative stress and DNA damage in human carcinogenesis. Mutat. Res..

[30] Cheng C., Ma H., Liu G., Fan S., Guo Z. (2022). Mechanism of cadmium exposure induced hepatotoxicity in the mud crab (*Scylla paramamosain*): Activation of oxidative stress and nrf2 signaling pathway. Antioxidants.

[31] Wang S., Xu J., Zheng J., Zhang X., Shao J., Zhao L., Hao J. (2020). Anti-inflammatory and antioxidant effects of acetyl-l-carnitine on *Atherosclerotic Rats*. Med. Sci. Monit..

[32] Wei X., Qi Y., Zhang X., Gu X., Cai H., Yang J., Zhang Y. (2015). ROS act as an upstream signal to mediate cadmium-induced mitophagy in mouse brain. NeuroToxicology.

[33] Durazzo A., Lucarini M., Nazhand A., Souto S.B., Silva A.M., Severino P., Souto E.B., Santini A. (2020). The nutraceutical value of carnitine and its use in dietary supplements. Molecules.

[34] Nishio T., Kishi R., Sato K., Sato K. (2022). Blue light exposure enhances oxidative stress, causes DNA damage, and induces apoptosis signaling in B16F1 melanoma cells. Mutat. Res. Genet. Toxicol. Environ. Mutagen..

[35] Pirinccioglu A.G., Gökalp D., Pirinccioglu M., Kizil G., Kizil M. (2010). Malondialdehyde (MDA) and protein carbonyl (PCO) levels as biomarkers of oxidative stress in subjects with familial hypercholesterolemia. Clin. Biochem..

[36] Yim W.W., Mizushima N. (2020). Lysosome biology in autophagy. Cell Discov..

[37] Zhou C., Song H., Feng J., Hu Z., Yang M.-J., Shi P., Li Y.-R., Guo Y.-J., Li H.-Z., Zhang T. (2022). Metabolomics and biochemical assays reveal the metabolic responses to hypo-salinity stress and osmoregulatory role of cAMP-PKA pathway in *Mercenaria mercenaria*. Comput. Struct. Biotechnol. J..

[38] Zhang A., Matsushita M., Zhang L., Wang H., Shi X., Gu H., Xia Z., Cui J.Y. (2021). Cadmium exposure modulates the gut-liver axis in an Alzheimer’s disease mouse model. Commun. Biol..

[39] Pi J., Li X., Zhang T., Li D. (2016). Effects of acute exposure to sublethal waterborne cadmium on energy homeostasis in silver carp (*Hypophthalmichthys molitrix*). Bull. Environ. Contam. Toxicol..

[40] Dando P.R. (1969). Lactate Metabolism in Fish. J. Mar. Biolog. Assoc. UK.

[41] El-Hattab A.W., Scaglia F. (2015). Disorders of carnitine biosynthesis and transport. Mol. Genet. Metab..

[42] Richard A.J., Stephens J.M. (2011). Emerging roles of JAK-STAT signaling pathways in adipocytes. Trends Endocrinol. Metab..

[43] Hong H., Lin X., Xu Y., Tong T., Zhang J., He H., Yang L., Lu Y., Zhou Z. (2022). Cadmium induces ferroptosis mediated inflammation by activating Gpx4/Ager/p65 axis in pancreatic β-cells. Sci. Total Environ..

[44] Selak M.A., Armour S.M., MacKenzie E.D., Boulahbel H., Watson D.G., Mansfield K.D., Pan Y., Simon M.C., Thompson C.B., Gottlieb E. (2005). Succinate links TCA cycle dysfunction to oncogenesis by inhibiting HIF-alpha prolyl hydroxylase. Cancer Cell.

[45] Lu Z., Xiao Z., Wu S., Song J., Peng X. (2022). Proteomic and metabolomic analysis on cadmium-induced mitochondrial toxicity in liver tissues of juvenile olive flounder *Paralichthys olivaceus*. Front. Mar. Sci..

[46] Ivanina A.V., Eilers S., Kurochkin I.O., Chung J.S., Techa S., Piontkivska H., Sokolov E.P., Sokolova I.M. (2010). Effects of cadmium exposure and intermittent anoxia on nitric oxide metabolism in eastern oysters, *Crassostrea virginica*. J. Exp. Biol..

[47] Zhang S., Wu L., Zhang J., Wang X., Yang X., Xin Y., Chen L., Li J., Niu P. (2023). Multi-omics analysis reveals Mn exposure affects ferroptosis pathway in zebrafish brain. Ecotoxicol. Environ. Saf..

[48] Krzak G., Willis C.M., Smith J.A., Pluchino S., Peruzzotti-Jametti L. (2021). Succinate receptor 1: An emerging regulator of myeloid cell function in inflammation. Trends Immunol..

[49] Wootton E.C., Dyrynda E.A., Pipe R.K., Ratcliffe N.A. (2003). Comparisons of PAH-induced immunomodulation in three bivalve molluscs. Aquat. Toxicol..

[50] Pena L.B., Barcia R.A., Azpilicueta C.E., Méndez A.A.E., Gallego S.M. (2012). Oxidative post translational modifications of proteins related to cell cycle are involved in cadmium toxicity in wheat seedlings. Plant Sci..

[51] Zhou Y., Dahms H.-U., Dong F., Jing W., Wang L. (2016). Immune-associated parameters and antioxidative responses to cadmium in the freshwater crab *Sinopotamon henanense*. Ecotoxicol. Environ. Saf..

