# Supplementary material for: Mechanisms of Gills Response to Cadmium Exposure in Greenfin Horse-Faced Filefish (Thamnaconus septentrionalis): Oxidative Stress, Immune Response, and Energy Metabolism
Source: Animals (Basel). 2024 Feb 7;14(4):561. doi: 10.3390/ani14040561 (PMC10886137; doi:10.3390/ani14040561)
Supplement: Supplementary file 1 [file animals-14-00561-s001.zip › animals-2849425-supplementary.pdf]

Supplementary Material

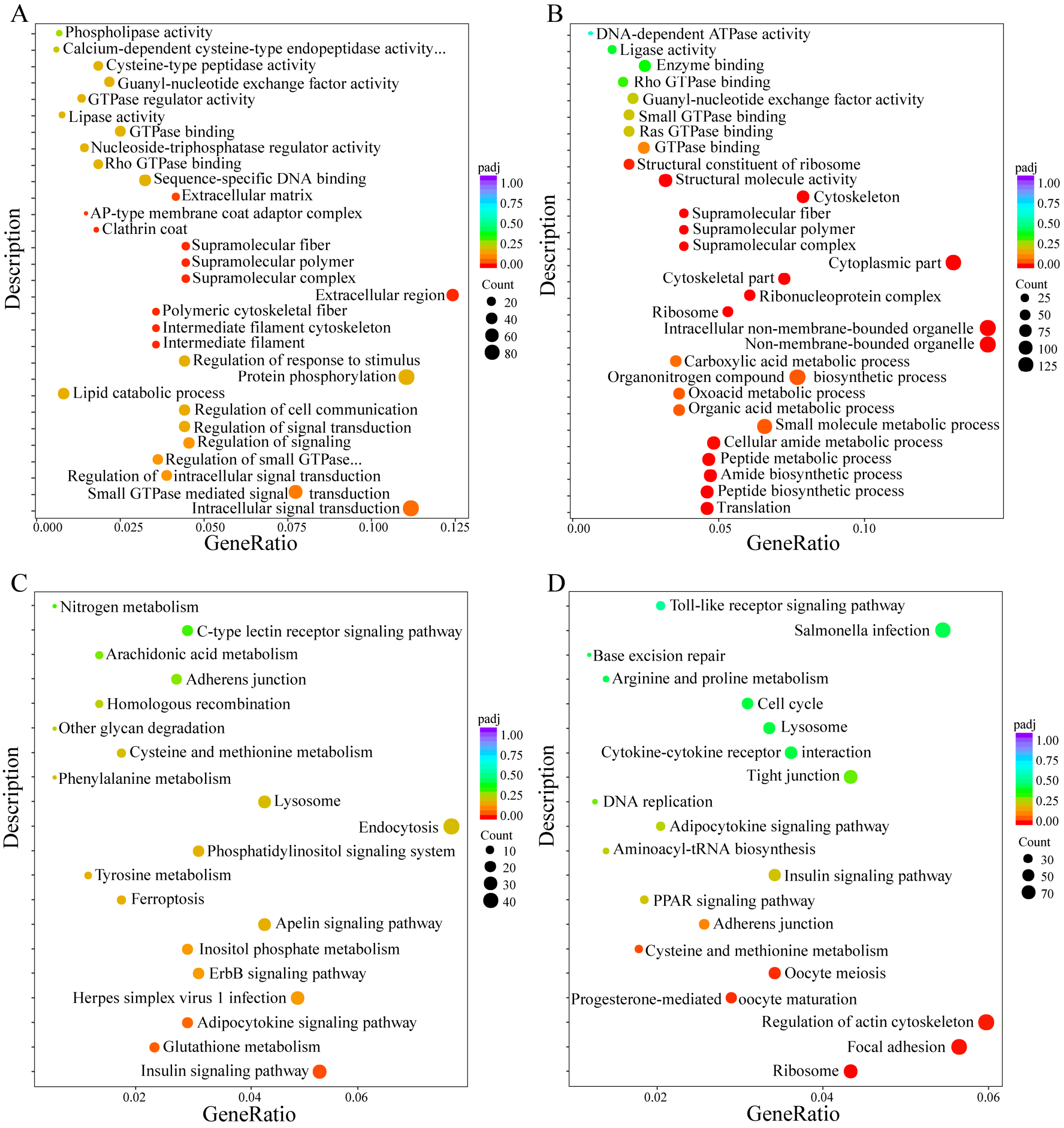

Figure S1. GO term and KEGG pathway analysis of filefish exposed to Cd. (A) The top 30 enriched GO terms based on DEGs of Cd_12h group. (B) The top 30 enriched GO terms based on DEGs of Cd_48h group. (C) The top 20 enriched KEGG pathways based on DEGs of Cd_12h group. (D) The top 20 enriched KEGG pathways based on DEGs of Cd_48h group.

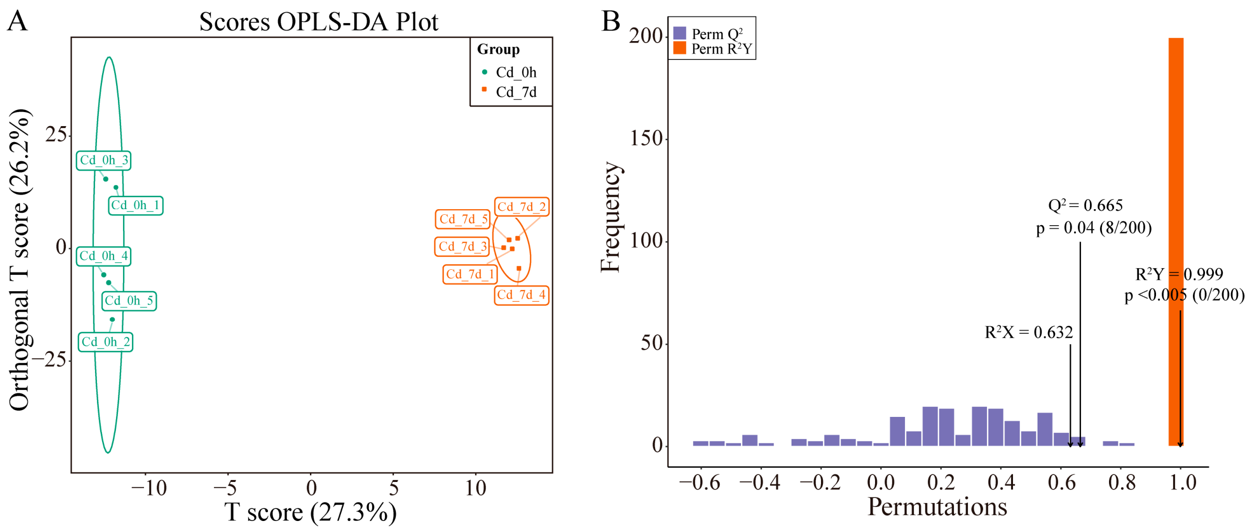

Figure S2. Multivariate statistical analysis of metabolites in filefish. (A) The OPLS-DA score plot. (B) The OPLS-DA model supervised and calculated the variables responsible for differences between groups. R2X and R2Y indicate the interpretation rate of X and Y matrix, respectively. Q2Y represents the prediction ability of the model. A value closer to 1 means that the model is more stable and reliable.

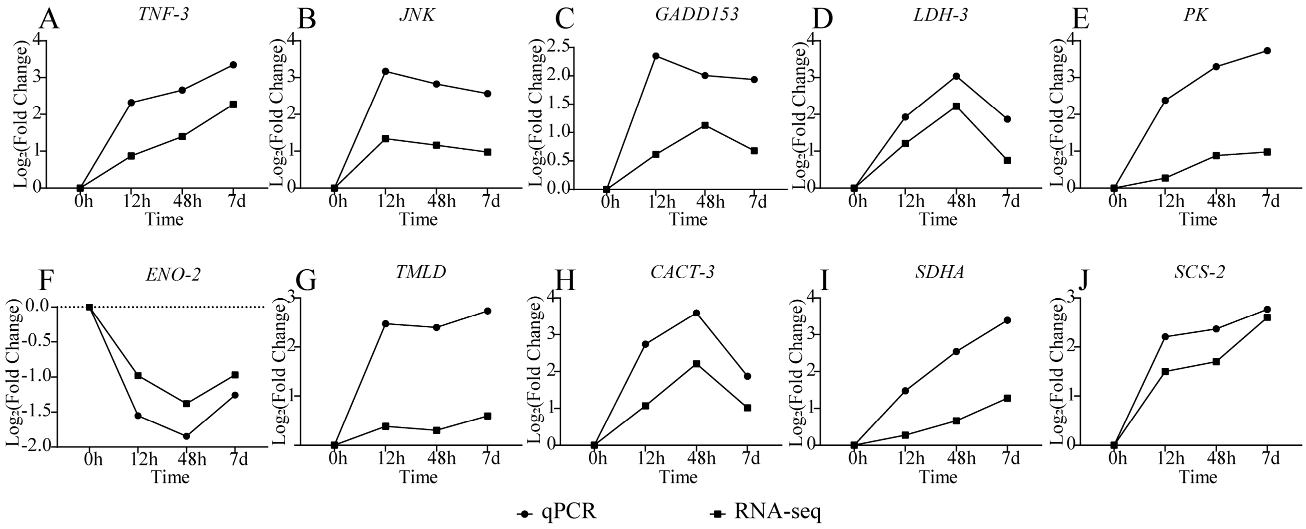

Figure S3. Comparison of DEGs expression levels between qPCR and RNA-Seq.

Table S1 Primer sequences for qPCR.

Gene name	Gene number	primer sequence (5'-3')
β-tublin	EVM0011833	Forward: GGCATCTATGAAGGCGACAGTTCC<br>Reverse: CCGTGGATGAAGTTGTCTGGTCTG
TNF-3	EVM0004334	Forward: AGGTCCAAACAAAGTGATGGGTCAT<br>Reverse: GCAGTGTGGCATGTATTGCTTGAAC
JNK	EVM0013119	Forward: CTTCCAGAATCAGACTCACGCCAAG<br>Reverse: GTCCATCAGCTCCATCACCAGGTA
GADD153	EVM0012728	Forward: TTCTCCTCCTCCTCCTCCTCCAT<br>Reverse: TCGTTCTGCTCCGTCAGTTCCT
LDH-3	EVM0008274	Forward: CGCTGATCGACGTGATGGAGGA<br>Reverse: ACCACCTTGGAGTTGGCTGTCA
PK	EVM0000571	Forward: CTCGCTCTGTGGACATGCTGAAG<br>Reverse: CTGTACTGGATGCTGCCTGGTTC
ENO-2	EVM0008813	Forward: CGCTGGAAACAGGCTGGCAAT<br>Reverse: TCTCCGACATTGGTGGCATCCT
TMLD	EVM0020645	Forward: ACCGCCACACTGACACATCCT<br>Reverse: TCAGCAGCGTAGAAGCCATCCA
CACT-3	EVM0005169	Forward: GGAGGTTAATAGCGGTGGAGGAAG<br>Reverse: GTTCATAAGACGAGAGGACGATGGC
SDHA	EVM0001147	Forward: GACAGGACAGGACACTCGCTACT<br>Reverse: GCACTCGCCATTCTCCATCAGG
SCS-2	EVM0009863	Forward: GCATGGCAGCTAATTTGGGTTTCAA<br>Reverse: CGGAACTCAGCGTTGTCGTCAA

Table S2 Summary of data quality in transcriptome sequencing.

Sample name	Raw reads[a]	Clean reads[b]	UMI reads[c]	Raw bases[d]	Clean bases[e]	Q20 (%)[f]	Q30 (%)[f]	GC (%)[g]	UMI Clean reads (%)[h]	Deduplicate Mapped UMI (%)[i]
Cd_0h_1	30183868	29609235	27366756	9.06G	8.88G	97.37	93.07	50.56	92.43	61.8
Cd_0h_2	19781637	19427749	17915811	5.93G	5.83G	97.42	93.12	51.03	92.22	63.27
Cd_0h_3	27144752	26709815	24964890	8.14G	8.01G	97.51	93.29	51.07	93.47	62.63
Cd_12h_1	22709096	22341188	20739445	6.81G	6.7G	97.54	93.35	51.12	92.83	62.59
Cd_12h_2	25293575	24964399	23525379	7.59G	7.49G	97.65	93.53	51.48	94.24	61.76
Cd_12h_3	21630144	21222711	19598548	6.49G	6.37G	97.65	93.51	50.54	92.35	60.03
Cd_48h_1	21545460	21196488	19847052	6.46G	6.36G	97.06	92.35	50.7	93.63	68.23
Cd_48h_2	23929383	23655848	22625830	7.18G	7.1G	97.29	92.85	51.94	95.65	66.59
Cd_48h_3	22432180	22123989	20835428	6.73G	6.64G	97.1	92.4	51.8	94.18	66.04
Cd_7d_1	25611464	25181598	23648478	7.68G	7.55G	97.3	92.9	51.98	93.91	66.67
Cd_7d_2	23364149	23008438	21622977	7.01G	6.9G	97.14	92.53	51.68	93.98	67.37
Cd_7d_3	28745774	28469690	27076213	8.62G	8.54G	97.24	92.63	51	95.11	63.03

[a] Raw reads：The count of original sequence data.
[b] Clean reads：The count of the filtered sequencing data.
[c] UMI reads：The number of reads that match the specific unique molecular identifiers pattern.
[d] Raw bases：The data size of Raw/Clean reads.
[e] Clean bases：The data size of Clean reads.
[f] Q20/Q30：The percentages of the bases with phred values larger than 20 and 30 in the total reads obtained by sequencing, respectively.
[g] GC：G and C are percentages of the four bases in clean reads.
[h] UMI Clean reads：Percentage of UMI reads in clean reads.
[i] Deduplicate Mapped UMI reads：The percentage of UMI reads after de duplication compared to the number of UMI reads on the reference genome.

Table S3 The ratio of sequencing reads mapping to genome in different samples.

Sample name	Cd_0h_1	Cd_0h_2	Cd_0h_3	Cd_12h_1	Cd_12h_2	Cd_12h_3	Cd_48h_1	Cd_48h_2	Cd_48h_3	Cd_7d_1	Cd_7d_2	Cd_7d_3
Total reads[a]	54733512	35831622	49929780	41478890	47050758	39197096	39694104	45251660	41670856	47296956	43245954	54152426
Total map[b]	46795464 (85.5%)	30040158 (83.84%)	43408418 (86.94%)	35619233 (85.87%)	41287368 (87.75%)	27776500 (70.86%)	34621561 (87.22%)	40032893 (88.47%)	36576751 (87.78%)	41243598 (87.2%)	37686353 (87.14%)	47815124 (88.3%)
Multiple mapped[c]	62583 (0.11%)	35568 (0.1%)	89815 (0.18%)	47434 (0.11%)	71387 (0.15%)	27806 (0.07%)	58837 (0.15%)	69612 (0.15%)	65489 (0.16%)	71179 (0.15%)	62510 (0.14%)	67377 (0.12%)
Uniquely mapped[d]	46732881 (85.38%)	30004590 (83.74%)	43318603 (86.76%)	35571799 (85.76%)	41215981 (87.6%)	27748694 (70.79%)	34562724 (87.07%)	39963281 (88.31%)	36511262 (87.62%)	41172419 (87.05%)	37623843 (87.0%)	47747747 (88.17%)
Read-1	23438702 (42.82%)	14972366 (41.79%)	21628388 (43.32%)	17752702 (42.8%)	20578388 (43.74%)	13834724 (35.3%)	17380300 (43.79%)	20073747 (44.36%)	18363444 (44.07%)	20666341 (43.69%)	18906787 (43.72%)	24002262 (44.32%)
Read-2	23294179 (42.56%)	15032224 (41.95%)	21690215 (43.44%)	17819097 (42.96%)	20637593 (43.86%)	13913970 (35.5%)	17182424 (43.29%)	19889534 (43.95%)	18147818 (43.55%)	20506078 (43.36%)	18717056 (43.28%)	23745485 (43.85%)
Reads map to '+'[e]	23326467 (42.62%)	14979466 (41.81%)	21622614 (43.31%)	17761251 (42.82%)	20583113 (43.75%)	13854959 (35.35%)	17254656 (43.47%)	19958803 (44.11%)	18235849 (43.76%)	20571653 (43.49%)	18794539 (43.46%)	23845490 (44.03%)
Reads map to '-'[e]	23406414 (42.76%)	15025124 (41.93%)	21695989 (43.45%)	17810548 (42.94%)	20632868 (43.85%)	13893735 (35.45%)	17308068 (43.6%)	20004478 (44.21%)	18275413 (43.86%)	20600766 (43.56%)	18829304 (43.54%)	23902257 (44.14%)
Non-splice reads	28035689 (51.22%)	18055111 (50.39%)	25793218 (51.66%)	20797933 (50.14%)	23556549 (50.07%)	15317221 (39.08%)	20908146 (52.67%)	22371115 (49.44%)	20867724 (50.08%)	22752717 (48.11%)	21503945 (49.72%)	26730345 (49.36%)
Splice reads	18697192 (34.16%)	11949479 (33.35%)	17525385 (35.1%)	14773866 (35.62%)	17659432 (37.53%)	12431473 (31.72%)	13654578 (34.4%)	17592166 (38.88%)	15643538 (37.54%)	1841970 2(38.94%)	16119898 (37.27%)	21017402 (38.81%)

The statistical results of the comparison are as follows:

[a] Total reads, the statistics of Clean reads after quality control.

[b] Total map: the statistics of Total reads that can be mapped to reference genome.

[c] Multiple mapped, the statistics of UMI reads with multiple alignment positions on the reference sequence.

[d] Uniquely mapped, the statistics of UMI reads with unique alignment position on the reference sequence.

[e] Reads map to '+'，Reads map to '-', the statistics of sequence alignment to positive and negative chains on the genome.

Table S4 Representative pathways related to Cd stress of filefish.

Function	signaling pathway	First Category	Second Category	Ratio(up-regulated genes/total genes)		
				Cd_12h vs Cd_0h	Cd_48h vs Cd_0h	Cd_7d vs Cd_0h
**Oxidative stress, oxidative damage and apoptosis**	Arachidonic acid metabolism	Metabolism	Lipid metabolism	5/9	7/13	12/16
	Fatty acid degradation	Metabolism	Lipid metabolism	3/5	6/14	9/11
	Fatty acid biosynthesis	Metabolism	Lipid metabolism	1/3	3/6	2/6
	Fatty acid metabolism	Metabolism	Lipid metabolism	3/5	9/11	8/14
	alpha-Linolenic acid metabolism	Metabolism	Lipid metabolism	0/1	1/2	3/3
	Linoleic acid metabolism	Metabolism	Lipid metabolism	1/2	2/2	5/5
	Lysosome	Cellular Processes	Transport and catabolism	10/24	20/48	23/38
	Autophagy - animal	Cellular Processes	Transport and catabolism	18/23	25/35	20/28
	Apoptosis	Cellular Processes	Cell growth and death	14/20	31/46	15/30
	Ferroptosis	Cellular Processes	Cell growth and death	9/11	7/11	10/15
	p53 signaling pathway	Cellular Processes	Cell growth and death	5/7	10/17	8/15
	MAPK signaling pathway	Environmental Information Processing	Signal transduction	28/40	60/82	34/58
**Energy metabolism**	Glycolysis / Gluconeogenesis	Metabolism	Carbohydrate metabolism	8/10	18/20	26/31
	Nitrogen metabolism	Metabolism	Energy metabolism	2/5	2/4	60/64
	Oxidative phosphorylation	Metabolism	Energy metabolism	3/4	7/15	3/5
	Alanine, aspartate and glutamate metabolism	Metabolism	Amino acid metabolism	5/8	7/13	7/13

Continued

Function	signaling pathway	First Category	Second Category	Ratio(up-regulated genes/total genes)		
				Cd_12h vs Cd_0h	Cd_48h vs Cd_0h	Cd_7d vs Cd_0h
**Immune response**	Citrate cycle (TCA cycle)	Metabolism	Carbohydrate metabolism	3/3	8/9	21/22
	Other glycan degradation	Metabolism	Glycan biosynthesis and metabolism	0/5	0/6	1/3
	Carbon metabolism	Metabolism	Global and overview maps	6/10	22/29	43/51
	Glycosaminoglycan degradation	Metabolism	Glycan biosynthesis and metabolism	0/2	2/8	2/2
	Cytokine-cytokine receptor interaction	Environmental Information Processing	Signaling molecules and interaction	5/17	30/52	23/48
	Regulation of actin cytoskeleton	Cellular Processes	Cell motility	22/34	65/88	43/69
	Toll-like receptor signaling pathway	Organismal Systems	Immune system	8/10	24/28	16/24
	Intestinal immune network for IgA production	Organismal Systems	Immune system	1/4	3/6	3/8
	C-type lectin receptor signaling pathway	Organismal Systems	Immune system	10/17	24/32	14/25
	Drug metabolism - cytochrome P450	Metabolism	Xenobiotics biodegradation and metabolism	3/6	4/9	5/9
	Metabolism of xenobiotics by cytochrome P450	Metabolism	Xenobiotics biodegradation and metabolism	4/7	5/9	3/7
	Arachidonic acid metabolism	Metabolism	Lipid metabolism	5/9	7/13	12/16
**Others**	2-Oxocarboxylic acid metabolism	Metabolism	Global and overview maps	~	3/3	6/6
	Pentose and glucuronate interconversions	Metabolism	Carbohydrate metabolism	0/2	4/6	6/9

Continued

Function	signaling pathway	First Category	Second Category	Ratio(up-regulated genes/total genes)		
				Cd_12h vs Cd_0h	Cd_48h vs Cd_0h	Cd_7d vs Cd_0h
	Glyoxylate and dicarboxylate metabolism	Metabolism	Carbohydrate metabolism	1/1	4/4	9/9
	Fanconi anemia pathway	Genetic Information Processing	Replication and repair	0/8	2/12	0/9
	ABC transporters	Environmental Information Processing	Membrane transport	2/5	6/9	5/9
	Adipocytokine signaling pathway	Organismal Systems	Endocrine system	12/17	17/28	14/24

Table S5 Significantly changed metabolites in filefish after 7 d of Cd stress.

Compounds	Class I	Class II	VIP	P-value	Log2FC	Type
Aniline	Alcohol and amines	Amines	1.30E+00	2.09E-02	4.17E-01	up
N1,N8-diacetylspermidine	Alcohol and amines	Amines	1.09E+00	4.74E-02	1.07E+00	up
p-Hydroxyphenylethanolamine	Alcohol and amines	Amines	1.67E+00	1.43E-02	-8.05E-01	down
3-Hydroxy-3-Methylpentane-1,5-Dioic Acid	Amino acid and Its metabolites	Amino acid derivatives	1.45E+00	1.11E-02	7.58E-01	up
Acetylvaline	Amino acid and Its metabolites	Amino acid derivatives	1.31E+00	2.41E-02	9.12E-01	up
Ala-Lys	Amino acid and Its metabolites	Amino acid derivatives	1.38E+00	6.92E-03	1.01E+00	up
DL-O-tyrosine	Amino acid and Its metabolites	Amino acid derivatives	1.76E+00	1.94E-04	3.42E-01	up
Gly-Phe	Amino acid and Its metabolites	Amino acid derivatives	1.48E+00	5.51E-03	1.31E+00	up
L-Carnosine	Amino acid and Its metabolites	Amino acid derivatives	1.37E+00	3.15E-02	1.10E+00	up
L-Histidinol	Amino acid and Its metabolites	Amino acid derivatives	1.36E+00	2.12E-02	9.44E-01	up
N-Acetyl-L-Leucine	Amino acid and Its metabolites	Amino acid derivatives	1.54E+00	8.13E-03	1.90E+00	up
N-Acetyl-L-alanine	Amino acid and Its metabolites	Amino acid derivatives	1.33E+00	3.31E-02	5.04E-01	up
N-Acetyl-L-methionine	Amino acid and Its metabolites	Amino acid derivatives	1.26E+00	2.91E-02	8.58E-01	up
N-Acetylthreonine	Amino acid and Its metabolites	Amino acid derivatives	1.54E+00	1.31E-02	9.90E-01	up
N-Methyl-D-Aspartic Acid	Amino acid and Its metabolites	Amino acid derivatives	1.58E+00	1.05E-02	-1.08E+00	down
N-acetylornithine	Amino acid and Its metabolites	Amino acid derivatives	1.53E+00	2.32E-02	7.37E-01	up
O-Acetyl-L-serine	Amino acid and Its metabolites	Amino acid derivatives	1.58E+00	1.05E-02	-1.08E+00	down
Phe-Hyp	Amino acid and Its metabolites	Amino acid derivatives	1.33E+00	2.04E-02	1.09E+00	up
S-(Methyl)glutathione	Amino acid and Its metabolites	Amino acid derivatives	1.51E+00	5.40E-03	1.03E+00	up
S-Allyl-L-cysteine	Amino acid and Its metabolites	Amino acid derivatives	1.47E+00	1.65E-02	1.62E+00	up
L-Lysine	Amino acid and Its metabolites	Amino acids	1.41E+00	1.71E-02	8.73E-01	up

Continued

Compounds	Class I	Class II	VIP	P-value	Log2FC	Type
L-Norleucine	Amino acid and Its metabolites	Amino acids	1.32E+00	1.84E-02	1.48E+00	up
L-Norvaline	Amino acid and Its metabolites	Amino acids	1.29E+00	3.67E-02	1.38E+00	up
L-Phenylalanine	Amino acid and Its metabolites	Amino acids	1.47E+00	1.34E-02	1.27E-01	up
L-Tryptophan	Amino acid and Its metabolites	Amino acids	1.47E+00	5.22E-03	1.07E+00	up
L-Valine	Amino acid and Its metabolites	Amino acids	1.29E+00	3.67E-02	1.38E+00	up
3-Aminoquinoline	Benzene and substituted derivatives	Benzene and substituted derivatives	1.47E+00	1.12E-02	6.07E-01	up
5-Acetylamino-6-formylamino-3-methyluracil	Benzene and substituted derivatives	Benzene and substituted derivatives	1.52E+00	2.38E-03	1.07E+00	up
Carnitine C11:0	FA	CAR	1.60E+00	6.38E-03	1.66E+00	up
Carnitine C12:1	FA	CAR	1.17E+00	3.12E-02	1.14E+00	up
Carnitine C13:0	FA	CAR	1.11E+00	2.54E-02	1.22E+00	up
Carnitine C13:1	FA	CAR	1.54E+00	5.15E-04	1.28E+00	up
Carnitine C14:1	FA	CAR	1.12E+00	4.90E-02	9.04E-01	up
Carnitine C14:2:DC	FA	CAR	1.35E+00	1.41E-02	1.23E+00	up
Carnitine C16-OH	FA	CAR	1.17E+00	3.73E-02	1.14E+00	up
Carnitine C18:4	FA	CAR	1.29E+00	3.30E-03	1.51E+00	up
Carnitine C3:0	FA	CAR	1.33E+00	9.79E-03	1.23E+00	up
Carnitine C4:0	FA	CAR	1.53E+00	1.68E-03	1.57E+00	up
Carnitine C5-OH	FA	CAR	1.37E+00	2.68E-02	1.39E+00	up
Carnitine C5:1	FA	CAR	1.48E+00	5.19E-03	1.50E+00	up
Carnitine C6:DC	FA	CAR	1.72E+00	8.25E-05	1.80E+00	up
Carnitine isoC4:0	FA	CAR	1.53E+00	1.68E-03	1.57E+00	up

Continued

Compounds	Class I	Class II	VIP	P-value	Log2FC	Type
Nicotinamide	CoEnzyme and vitamins	CoEnzyme and vitamins	1.35E+00	2.95E-02	1.20E+00	up
Pantothenate	CoEnzyme and vitamins	CoEnzyme and vitamins	1.30E+00	2.16E-02	1.00E+00	up
Pyridoxine 5'-Phosphate	CoEnzyme and vitamins	CoEnzyme and vitamins	1.48E+00	2.66E-02	1.01E+00	up
Riboflavin	CoEnzyme and vitamins	CoEnzyme and vitamins	1.44E+00	3.78E-02	1.30E+00	up
8,15-Dihete	FA	FFA	1.48E+00	2.00E-02	9.17E-01	up
FFA(15:1)	FA	FFA	1.27E+00	2.45E-02	-5.78E-01	down
FFA(16:1)	FA	FFA	1.27E+00	3.59E-02	8.40E-01	up
FFA(22:5)	FA	FFA	1.32E+00	4.41E-02	9.79E-01	up
FFA(22:7)	FA	FFA	1.33E+00	2.64E-02	1.04E+00	up
3-Amino-2-piperidinone	Heterocyclic compounds	Heterocyclic compounds	1.33E+00	2.07E-02	6.55E-01	up
Indoleacetaldehyde	Heterocyclic compounds	Indole and Its derivatives	1.55E+00	1.35E-03	1.08E+00	up
1-arachidonoyl-2-hydroxy-sn-glycero-3-phosphate	GP	LPA	1.57E+00	3.38E-03	1.63E+00	up
LPC(18:1/0:0)	GP	LPC	1.12E+00	4.15E-02	1.37E+00	up
LPC(O-16:0)	GP	LPC-O	1.49E+00	7.89E-03	1.33E+00	up
LPE(O-18:2)	GP	LPE	1.30E+00	4.44E-02	1.19E+00	up
MG(20:5/0:0/0:0)	GL	MG	1.65E+00	6.54E-04	1.72E+00	up
2'-Aenylic Acid	Nucleotide and Its metabolites	Nucleotide and Its metabolites	1.63E+00	1.09E-03	1.54E+00	up
2-Aminopurine	Nucleotide and Its metabolites	Nucleotide and Its metabolites	1.38E+00	1.67E-02	1.14E+00	up
3'-Adenylic acid	Nucleotide and Its metabolites	Nucleotide and Its metabolites	1.63E+00	1.09E-03	1.54E+00	up
5'-Adenylyl sulfate(APS)	Nucleotide and Its metabolites	Nucleotide and Its metabolites	1.48E+00	2.22E-02	1.75E+00	up
5'-Deoxy-5'-(Methylthio) Adenosine	Nucleotide and Its metabolites	Nucleotide and Its metabolites	1.26E+00	2.34E-02	1.01E+00	up

Continued

Compounds	Class I	Class II	VIP	P-value	Log2FC	Type
5'-Deoxy-5'-fluoroadenosine	Nucleotide and Its metabolites	Nucleotide and Its metabolites	1.29E+00	4.68E-02	1.08E+00	up
ADP-ribose	Nucleotide and Its metabolites	Nucleotide and Its metabolites	1.44E+00	2.43E-02	9.58E-01	up
Adenosine	Nucleotide and Its metabolites	Nucleotide and Its metabolites	1.30E+00	4.08E-02	1.11E+00	up
Adenosine 5'-Diphosphate（ADP）	Nucleotide and Its metabolites	Nucleotide and Its metabolites	1.57E+00	2.19E-02	1.86E+00	up
Adenosine 5'-Monophosphate	Nucleotide and Its metabolites	Nucleotide and Its metabolites	1.65E+00	1.39E-03	1.53E+00	up
Arainosine	Nucleotide and Its metabolites	Nucleotide and Its metabolites	1.26E+00	4.46E-02	1.04E+00	up
Cyclic Amp	Nucleotide and Its metabolites	Nucleotide and Its metabolites	1.53E+00	4.58E-03	1.21E+00	up
Deoxycytidine	Nucleotide and Its metabolites	Nucleotide and Its metabolites	1.66E+00	2.89E-03	-6.49E-01	down
Deoxyguanosine 5'-monophosphate(dGMP)	Nucleotide and Its metabolites	Nucleotide and Its metabolites	1.71E+00	2.07E-03	1.99E+00	up
GDP-L-fucose	Nucleotide and Its metabolites	Nucleotide and Its metabolites	1.58E+00	1.31E-02	1.66E+00	up
Inosine 5'-monophosphate	Nucleotide and Its metabolites	Nucleotide and Its metabolites	1.63E+00	2.07E-03	9.62E-01	up
N6-Succinyl Adenosine	Nucleotide and Its metabolites	Nucleotide and Its metabolites	1.45E+00	9.38E-03	1.09E+00	up
Thioguanine	Nucleotide and Its metabolites	Nucleotide and Its metabolites	1.38E+00	3.47E-02	-6.71E-01	down
2-Hydroxy-2-Methyl Butyric acid	Organic acid and Its derivatives	Organic acid and Its derivatives	1.31E+00	3.93E-02	7.75E-01	up
2-Methyllactic acid	Organic acid and Its derivatives	Organic acid and Its derivatives	1.43E+00	7.51E-03	1.07E+00	up
2-amino-4-oxovaleric acid	Organic acid and Its derivatives	Organic acid and Its derivatives	1.33E+00	3.31E-02	5.04E-01	up
2-methyl citric acid	Organic acid and Its derivatives	Organic acid and Its derivatives	1.34E+00	3.51E-02	1.32E+00	up
8-Aminooctanoic Acid	Organic acid and Its derivatives	Organic acid and Its derivatives	1.44E+00	5.40E-03	8.49E-01	up
Aminomalonic Acid	Organic acid and Its derivatives	Organic acid and Its derivatives	1.51E+00	8.20E-03	4.38E-01	up
D-Malic acid	Organic acid and Its derivatives	Organic acid and Its derivatives	1.31E+00	1.90E-02	1.15E+00	up
Iminodiacetic acid	Organic acid and Its derivatives	Organic acid and Its derivatives	1.55E+00	1.56E-02	-3.05E-01	down

Continued

Compounds	Class I	Class II	VIP	P-value	Log2FC	Type
L-Lactic Acid	Organic acid and Its derivatives	Organic acid and Its derivatives	1.32E+00	1.10E-02	9.83E-01	up
Malonic acid	Organic acid and Its derivatives	Organic acid and Its derivatives	1.40E+00	1.73E-02	6.57E-01	up
Methylmalonic Acid	Organic acid and Its derivatives	Organic acid and Its derivatives	1.58E+00	1.61E-03	1.31E+00	up
Salicylic acid	Organic acid and Its derivatives	Organic acid and Its derivatives	1.23E+00	4.78E-02	1.37E-01	up
Succinic Acid	Organic acid and Its derivatives	Organic acid and Its derivatives	1.58E+00	1.61E-03	1.31E+00	up
Tetradecyl phosphonic acid	Organic acid and Its derivatives	Organic acid and Its derivatives	1.45E+00	9.41E-03	4.51E-01	up
α-Hydroxyglutaric Acid (sodium salt)	Organic acid and Its derivatives	Organic acid and Its derivatives	1.31E+00	3.74E-02	7.07E-01	up
2-Hexadecanoylthio-1-ethylphosphorylcholine	FA	Others	1.37E+00	1.57E-02	9.16E-01	up
Heterodendrin	Others	Others	1.43E+00	8.71E-03	1.28E+00	up
methylcarbamyl PAF	FA	Others	1.38E+00	1.46E-02	8.94E-01	up
(±)17-HDHA	FA	Oxidized lipids	1.46E+00	3.21E-03	1.27E+00	up
(±)18-HEPE	FA	Oxidized lipids	1.46E+00	4.91E-02	1.63E+00	up
(±)9-HETE	FA	Oxidized lipids	1.68E+00	1.31E-02	1.65E+00	up
14(S)-HDHA	FA	Oxidized lipids	1.65E+00	2.36E-04	1.54E+00	up
16-HDoHE	FA	Oxidized lipids	1.69E+00	9.40E-04	1.59E+00	up
8-HDoHE	FA	Oxidized lipids	1.62E+00	1.50E-02	1.92E+00	up
Pyrocatechol	Benzene and substituted derivatives	Phenolics	1.35E+00	2.62E-02	-1.29E-01	down
2-Amino-3-phosphonopropionic acid	Organic acid and Its derivatives	Phosphoric acids	1.46E+00	7.44E-03	-1.12E+00	down
Biotinamide	Alcohol and amines	Polyamines	1.49E+00	6.88E-03	1.56E+00	up
N-Acetylcadaverine	Alcohol and amines	Polyamines	1.22E+00	3.59E-02	-3.40E-01	down
gamma-Glu-Phe	Amino acid and Its metabolites	polypeptide	1.43E+00	1.47E-02	1.25E+00	up

Continued

Compounds	Class I	Class II	VIP	P-value	Log2FC	Type
Ala-Pro	Amino acid and Its metabolites	Small Peptide	1.34E+00	3.57E-02	-3.90E-01	down
Arg-Gly	Amino acid and Its metabolites	Small Peptide	1.91E+00	8.14E-04	1.45E+01	up
Asn-Ser	Amino acid and Its metabolites	Small Peptide	1.29E+00	4.05E-02	7.97E-01	up
Cys-Glu	Amino acid and Its metabolites	Small Peptide	1.42E+00	3.57E-02	-5.67E-01	down
Glu-Gly	Amino acid and Its metabolites	Small Peptide	1.53E+00	7.33E-03	8.27E-01	up
Glu-Ile	Amino acid and Its metabolites	Small Peptide	1.68E+00	1.29E-03	1.23E+00	up
Glu-Leu	Amino acid and Its metabolites	Small Peptide	1.38E+00	1.06E-02	1.07E+00	up
Glu-Met	Amino acid and Its metabolites	Small Peptide	1.17E+00	3.51E-02	9.75E-01	up
Glu-Phe-Ala	Amino acid and Its metabolites	Small Peptide	1.50E+00	1.72E-03	1.28E+00	up
Glu-Tyr	Amino acid and Its metabolites	Small Peptide	1.31E+00	1.56E-02	1.14E+00	up
Glu-Val	Amino acid and Its metabolites	Small Peptide	1.34E+00	1.51E-02	1.19E+00	up
Gly-Arg	Amino acid and Its metabolites	Small Peptide	1.91E+00	8.14E-04	1.45E+01	up
Gly-Glu	Amino acid and Its metabolites	Small Peptide	1.53E+00	7.33E-03	8.27E-01	up
Gly-Ile	Amino acid and Its metabolites	Small Peptide	1.45E+00	4.06E-02	1.29E+00	up
Ile-Glu	Amino acid and Its metabolites	Small Peptide	1.68E+00	1.29E-03	1.23E+00	up
Ile-Ile	Amino acid and Its metabolites	Small Peptide	1.69E+00	6.60E-04	1.14E+00	up
Ile-Leu	Amino acid and Its metabolites	Small Peptide	1.64E+00	1.82E-04	1.27E+00	up
Ile-Ser	Amino acid and Its metabolites	Small Peptide	1.42E+00	9.88E-03	1.01E+00	up
Leu-Glu	Amino acid and Its metabolites	Small Peptide	1.68E+00	1.29E-03	1.23E+00	up
Leu-Gly	Amino acid and Its metabolites	Small Peptide	1.52E+00	2.91E-02	1.37E+00	up
Leu-Leu	Amino acid and Its metabolites	Small Peptide	1.64E+00	1.82E-04	1.27E+00	up
Lys-Ala	Amino acid and Its metabolites	Small Peptide	1.38E+00	6.92E-03	1.01E+00	up

Continued

Compounds	Class I	Class II	VIP	P-value	Log2FC	Type
Lys-Gly	Amino acid and Its metabolites	Small Peptide	1.39E+00	2.57E-02	6.40E-01	up
Lys-Phe	Amino acid and Its metabolites	Small Peptide	1.61E+00	2.61E-03	6.30E-01	up
Met-Glu	Amino acid and Its metabolites	Small Peptide	1.39E+00	1.83E-02	1.11E+00	up
Phe-Ala	Amino acid and Its metabolites	Small Peptide	1.76E+00	2.69E-05	2.12E+00	up
Phe-Asp	Amino acid and Its metabolites	Small Peptide	1.54E+00	3.36E-02	1.87E+00	up
Phe-Gly	Amino acid and Its metabolites	Small Peptide	1.54E+00	8.00E-03	1.12E+00	up
Pro-Ala	Amino acid and Its metabolites	Small Peptide	1.23E+00	4.04E-02	-5.39E-01	down
Pro-Asn	Amino acid and Its metabolites	Small Peptide	1.41E+00	4.39E-02	1.46E+00	up
Pro-Leu	Amino acid and Its metabolites	Small Peptide	1.55E+00	2.59E-03	1.10E+00	up
Proline-Hydroxyproline	Amino acid and Its metabolites	Small Peptide	1.45E+00	6.47E-03	1.06E+00	up
Ser-Gln	Amino acid and Its metabolites	Small Peptide	1.18E+00	3.82E-02	7.75E-01	up
Ser-Phe	Amino acid and Its metabolites	Small Peptide	1.58E+00	1.37E-03	1.13E+00	up
Tyr-Leu	Amino acid and Its metabolites	Small Peptide	1.80E+00	9.41E-04	1.73E+00	up
Tyr-Pro	Amino acid and Its metabolites	Small Peptide	1.60E+00	1.64E-03	1.26E+00	up
Tyr-Thr	Amino acid and Its metabolites	Small Peptide	1.57E+00	1.69E-02	1.45E+00	up
Val-Ile	Amino acid and Its metabolites	Small Peptide	1.70E+00	1.13E-02	1.72E+00	up
Val-Leu	Amino acid and Its metabolites	Small Peptide	1.70E+00	1.13E-02	1.72E+00	up
D-Saccharic Acid 1,4-lactone (hydrate)	Carbohydrates and Its metabolites	Sugars	1.23E+00	4.29E-02	1.13E+00	up
Tryptamine	Tryptamines,Cholines,Pigments	Tryptamines	1.54E+00	2.30E-03	9.55E-01	up
